# Study Protocol – Improving Access to Kidney Transplants (IMPAKT): A detailed account of a qualitative study investigating barriers to transplant for Australian Indigenous people with end-stage kidney disease

**DOI:** 10.1186/1472-6963-8-31

**Published:** 2008-02-04

**Authors:** Jeannie Devitt, Alan Cass, Joan Cunningham, Cilla Preece, Kate Anderson, Paul Snelling

**Affiliations:** 1 The George Institute for International Health, PO Box M201, Missenden Road Sydney, NSW, 2050 Australia & Cooperative Research Centre for Aboriginal Health, Darwin, Australia; 2 The George Institute for International Health, PO Box M201, Missenden Road Sydney, NSW 2050, Australia; 3 Menzies School of Health Research and Institute of Advanced Studies, Charles Darwin University, PO Box 41096, Casuarina, NT 0811, Australia; 4 Cairns Diabetes Centre, Queensland Department of Health, PO Box 902, Cairns Qld 4870, Australia; 5 Statewide Renal Services, Royal Prince Alfred Hospital, Sydney, NSW 2050, Australia

## Abstract

**Background:**

Indigenous Australians are slightly more than 2% of the total Australian population however, in recent years they have comprised between 6 and 10% of new patients beginning treatment for end-stage kidney disease (ESKD).  Although transplant is considered the optimal form of treatment for many ESKD patients there is a pronounced disparity between the rates at which Indigenous ESKD patients receive transplants compared with their non-Indigenous counterparts. The IMPAKT (Improving Access to Kidney Transplants) Interview study investigated reasons for this disparity through a large scale, in-depth interview study involving patients, nephrologists and key decision-making staff at selected Australian transplant and dialysis sites.

**Methods:**

The design and conduct of the study reflected the multi-disciplinary membership of the core IMPAKT team.  Promoting a participatory ethos, IMPAKT established partnerships with a network of hospital transplant units and hospital dialysis treatment centres that provide treatment to the vast majority of Indigenous patients across Australia.  Under their auspices, the IMPAKT team conducted in-depth interviews in 26 treatment/service centres located in metropolitan, regional and remote Australia.  Peer interviewing supported the engagement of Indigenous patients (146), and nephrologists (19).  In total IMPAKT spoke with Indigenous and non-Indigenous patients (241), key renal nursing and other (non-specialist) staff (95) and a small number of relevant others (28). Data analysis was supported by QSR software.  At each site, IMPAKT also documented educational programs and resources, mapped an hypothetical ‘patient journey’ to transplant through the local system and observed patient care and treatment routines.

**Discussion:**

The national scope, inter-disciplinary approach and use of qualitative methods in an investigation of a significant health inequality affecting Indigenous people is, we believe, an Australian first.  An exceptionally large cohort of Indigenous participants provided evaluative comment on their health services in relation to dialysis and transplant. Additionally, the data includes extensive parallel commentary from a cohort of specialists, nurses and other staff.  The study considers a ‘patient journey’ to transplant within a diverse range of Australian treatment centre/workplace settings.  The IMPAKT Interview study protocol may contribute to improvements in multi-disciplinary, flexible design health services research with hard to reach or vulnerable populations in Australia and elsewhere.

## Background

Aboriginal and Torres Strait Islander people, the Indigenous Australians, now number around 460,000 people or slightly more than 2% of the total Australian population [1]. However they currently comprise between 6 and 10% [2] of new patients beginning treatment for end stage kidney disease (ESKD). Disproportionate levels of chronic kidney disease (CKD) and related conditions (diabetes and cardio-vascular disease) are evident among Indigenous peoples in other affluent nations including Canada, New Zealand and the USA [3,4]. Within Australia, kidney disease is more widespread among Indigenous people living in regional and remote areas [5]. End-stage kidney disease (ESKD), the most severe manifestation of CKD, usually occurs following many years of progressive loss of kidney function. When a person has ESKD, they require ongoing dialysis or a kidney transplant to stay alive.

Although transplant is considered the optimal form of treatment for many ESKD patients [6,7] there is a pronounced disparity in access to kidney transplants; with Indigenous Australians receiving transplants at approximately one third the rate of other patients. Moreover, those that do receive transplants have waited longer for them. Indigenous patients are both less likely to receive a transplant and less likely to be wait-listed for a transplant [8].

Barriers to higher Indigenous transplant rates may be related to system level factors including criteria (implicit or explicit) used for organ allocation [9-11]; reduced likelihood of referral for transplant evaluation and failure to complete the work-up requirements [12]. Individual-level factors may include race/ethnicity where members of minority groups are systematically disadvantaged by transplant allocation systems [12], residence location in relation to transplant units [13], challenges in adopting complex treatment requirements [14,15], poor health with high levels of co-morbid conditions [16], and reduced options for living kidney donation (LKD) due to high prevalence of risk factors for kidney disease in the Indigenous populations [17].

There is evidence that miscommunication and/or inadequate communication significantly affects treatment outcomes [18]. Ineffective information and education processes for Indigenous patients who are non-English speaking, often poorly literate and unfamiliar with institutional health care environments have been linked to patient isolation, reduced engagement in treatment management and associated issues of 'non-compliance'[19]. Although specific Australian research has not been conducted, many Indigenous kidney patients are likely to have limited 'health literacy' where that is defined as:

*The degree to which individuals have the capacity to obtain, process, and understand basic health information and services needed to make appropriate health decisions *[20].

A growing international literature indicates that limited health literacy is both highly prevalent and consistently associated with education, ethnicity and age [21-25]. Health literacy has a "measurable impact on numerous intermediate factors affecting health outcomes" and is not accounted for in standard patient education and care programs [26].

The IMPAKT program – Improving Access to Kidney Transplants – was established to further investigate the nature of barriers impeding rates of kidney transplant for Indigenous Australians. IMPAKT draws on evidence of continuing inequalities in access to kidney transplant [8,12] and more broadly, on literature documenting disparities in healthcare associated with race and/or ethnicity [27-29]. This paper describes the design, planning and conduct of the IMPAKT 'Interview' study – one of the larger components of the overall research program.

### The IMPAKT research program

The IMPAKT research program had five key objectives:

1) To assess the impact of medical and socio-demographic variables, especially Indigenous status, on the likelihood of being deemed medically suitable for renal transplant;

2) To evaluate the appropriateness, accessibility and effectiveness of patient education programs about renal transplant for Indigenous patients;

3) To identify systemic barriers to completing the essential steps towards transplant;

4) To examine the effect of current deceased-organ allocation algorithms upon Indigenous patients' access to transplant, and to model alternative allocations; and

5) To collaborate with health service providers to investigate current practices and to improve the efficiency and equity of transplant services.

**Figure 1:**
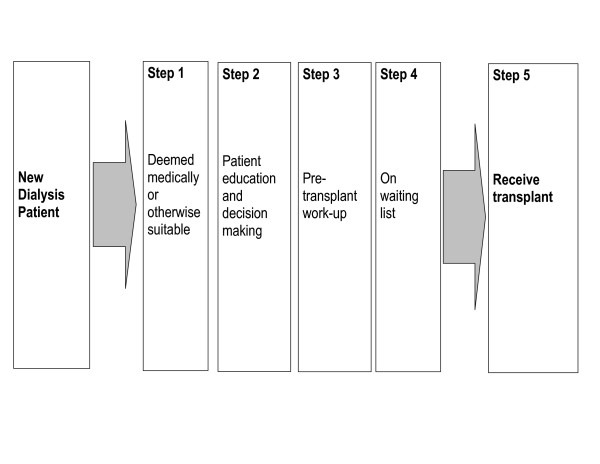
**The 'Steps' model underpinning the IMPAKT research program**. All patients need to move through these steps to achieve a transplant (adapted from Alexander and Sehgal 1998)

A model delineating the essential sequence of 'steps' for any dialysis patient to receive a transplant [30] provided a conceptual framework for the research program. The IMPAKT adaptation of this model (Figure 1) proposed five steps making up the path to transplant for a new dialysis patient: being deemed medically suitable; becoming informed and making appropriate decisions; completing the preparation or 'workup' for transplant; being placed on the waiting list; receiving a transplant. We refer to this as the 'Steps' model.

One or more of the five 'steps' provided a focus for the sub-studies that comprised the full IMPAKT research program. We now see the Steps model itself as requiring important revisions – a subject of future publications – but it continues to be a productive framework for the overall program. The sub-studies included:

a) A survey of Australian nephrologists to investigate the significance of selected variables including ethnicity, in their assessment of patients' medical suitability for transplant [17,31].

b) A qualitative study investigating potential barriers within the health systems and services. This sub-study, referred to as the 'Interview study' is the subject of this paper.

c) A review of clinical practice guidelines for determining recipient suitability for kidney transplant [32].

d) As the research proceeded it became evident that a deeper understanding of the role of multiple systemic barriers would be more productive in terms of potential change than modeling alternative kidney allocation systems.

The final objective of the study – considering ways to improve the processes through which all suitable patients may move to transplant – is on-going.

## Methods

### Design and planning

The Steps model underpinned the Interview study but not through a straightforward correspondence between data collection and 'step'. Notwithstanding the conceptual reality of each of the identifiable 'steps' in patients' progress to transplant, on the ground in Australian renal units, such a pathway is neither readily apparent, nor necessarily the most significant influence on events. With the exception of those few who are pre-emptively transplanted (they receive a transplant before commencing dialysis treatment), potential transplant recipients are undergoing dialysis. A person with ESKD is involved in an intensive treatment regime, in which transplant decision-making is essentially one among many *inter-related *decisions regarding treatment [33]. This study explored how local decision-making pathways and conditions, over the long period of treatment necessitated by ESKD, might take a patient either towards, or away from, transplant. Qualitative or 'flexible design' research [34] is well suited to this kind of research, exploring *why *people do what they do, and how they perceive the central issues.

The IMPAKT Interview study investigated three related questions:

1) How, and how effectively, are Indigenous ESKD patients informed and educated about their illness and treatment options, including transplant?

2) What factors, processes and conditions shape decision-making in relation to transplant options for ESKD patients, in particular for Indigenous patients?

3) What barriers prevent Indigenous ESKD patients receiving transplants at rates comparable to their non-Indigenous counterparts?

**Figure 2:**
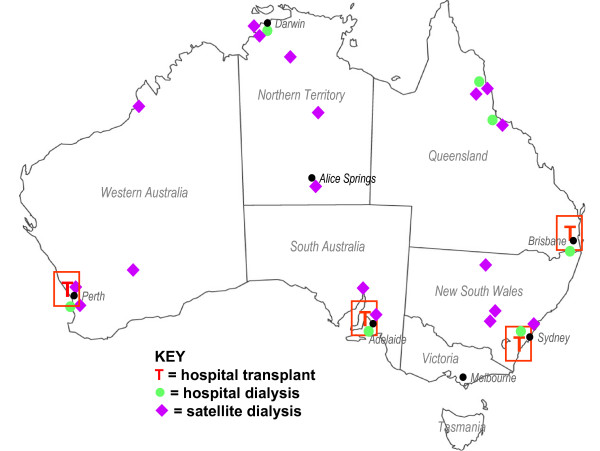
IMPAKT interview sites.

The remainder of this paper is a detailed account of the methods used. In brief, we sought to answer the questions by interviewing key stakeholder groups at selected treatment sites (i.e. transplant and dialysis sites). The stakeholder groups comprised, on the one hand, patients – particularly Indigenous patients – and, on the other, staff with roles or tasks related to each of the identified five steps, including nephrologists, transplant coordinators, patient educators and so on. In total, we interviewed 355 patients and staff (Table 1) and 28 other relevant people (carers, family members and so on) at locations across Australia (Figure 2). At each site, IMPAKT carried out three additional exercises: first, documenting educational programs and resources; second, facilitating an interactive activity mapping a hypothetical 'patient journey' from diagnosis to transplant through that local system; and third, observing interactions, patient care, and treatment routines during field visits to service centres. Although referred to as the 'Interview' Study, this additional qualitative research component [35] including observation; interview recording and transcription; and analysis of texts and documents.

**Table 1:** Service centres, showing size, setting, patient/staff interviewee numbers and Indigenous status

	**Service Centres**				**Interviewees**			
**State**	**Site**	**Type**	**No. of chairs**	**ARIA rank**	**Patients**			**Staff**^3^

					**I**^2^	**non-I**^2^	**total**	

NSW	1	hospital + tx unit	11	HA	1	8	9	4
	2	hospital	12	HA	0	7	7	0
	3	hospital	33*	HA	0	2	2	0
	4	satellite unit	10	A	5	10	15	5
	5	satellite unit	3	R	2	0	2	0
	6	satellite unit	3	A	2	1	3	0
					**10**	**28**	**38**	**9**
WA	7	hospital + tx unit	12*	HA	1	5	6	8
	8	satellite unit	20	HA	7	8	15	2
	9	satellite unit	12	HA	5	5	10	4
	10	satellite unit	6	MA	10	1	11	11
	11	satellite unit	10	R	22	1	23	11
					**45**	**20**	**65**	**36**
QLD	12	hospital + tx unit		HA	5	8	13	9
	13	hospital	13*	MA	1	1	2	9
	14	satellite unit	8	MA	13	5	18	0
	15	satellite unit	8	MA	6	5	11	4
	16	hospital		MA	6	1	7	8
	17	satellite unit	8	MA	7	5	12	1
					**38**	**25**	**63**	**31**
SA	18	hospital + tx unit	12*	HA	1	4	5	7
	19	satellite unit	16	HA	2	5	7	2
	20	satellite unit	10	MA	7	7	14	6
					**10**	**16**	**26**	**15**
NT	21	hospital	6	MA	0	1	1	7
	22	satellite unit	22*	MA	6	2	8	4
	23	satellite unit	8	MA	6	0	6	1
	24	satellite unit	4	R	5	1	6	1
	25	satellite unit	8	VR	9	0	9	3
	26	satellite unit	26	R	17	2	19	7
					**43**	**6**	**49**	**23**

**Total**					**146**	**95**	**241**	**114**

The ARIA scale has 5 classes of remoteness: HA = Highly Accessible; A = Accessible; MA = Moderately Accessible; R = Remote; VR = Very Remote

* includes chairs allocated for teaching home haemodialysis patients

2: I = Indigenous; non-I = non-Indigenous

3: 'staff' in this table includes nephrologists

Two key considerations shaped the overall study design:

• maximizing the number of Aboriginal/Torres Strait Islander participants, a 'hard-to-reach'/vulnerable population; and,

• maximizing the range of settings in which renal care is provided (urban, regional, remote, private, public, etc).

Achieving the first of these had both methodological and logistical ramifications that are described below. The second entailed establishing a research partnership with eight metropolitan and regional hospitals across Australia that, together, treat the vast majority of Aboriginal and Torres Strait Islander ESKD patients. Additionally, and under the auspices of the eight participating hospitals, we conducted research with patients and staff in a further 18 dialysis service centres at locations across the country (Table 1, Figure 2).

#### Participants and sampling

The Interview study used a purposive or maximum diversity sampling strategy, which operated at several levels:

#### Systemic aspects

In relation to systemic aspects of care (processes, programs, resources etc) the objectives were to maximise:

• the involvement of transplant units treating the greatest number of Aboriginal/Torres Strait Islander patients;

• the range of settings in which care is provided (urban, regional, remote, private, public etc);

• the involvement of key decision-makers; and

• involvement of Indigenous staff.

#### Patients

To explore the experiences of Aboriginal and Torres Strait Islander patients, objectives at each site were to:

• maximize the number of Aboriginal/Torres Strait Islander patient participants;

• maximize the range of treatments being undertaken (home and centre-based haemodialysis, peritoneal dialysis, transplant);

• include equal numbers of male and female participants;

• focus on people between the ages of 18 and 65 years;

• seek people who had begun dialysis relatively recently (last 5 years); and

• maximize involvement of patients who were non-English speakers.

There were two further patient-level considerations:

• including non-indigenous patients at each site; and

• maintaining a proportionality at the level of the site and the state between numbers of new Indigenous patients starting on dialysis and numbers interviewed.

A matrix was used to both map the range of (patient) viewpoints and think through the practical & logistical implications of achieving that level of diversity. With some gaps, the study ultimately achieved a high level of diversity across the patient participant categories (Table 2).

**Table 2:** Matrix summarising diversity achieved in patient interviewees

**Patient Characteristics**	**Treatment Types**					
	**Haemodialysis**^1^		**Peritoneal dialysis**		**Transplant**	

	**Indigenous**	**Non-Indigenous**	**Indigenous**	**Non-Indigenous**	**Indigenous**	**Non-Indigenous**

Age range (18–65 yrs)	✓	✓	✓	✓	✓	✓
Male + female	✓	✓	✓	✓	**x**	✓
English + non-English^2^	✓	✓	✓	**x**	✓	✓
Centre care + self care	✓	✓	**NA**	**NA**	**NA**	**NA**
Relatively recent start on dialysis	✓	✓	✓	✓	✓	✓
On/not on Tx waiting list	✓	✓	**x**	✓	**NA**	**NA**
Urban care setting	✓	✓	✓	✓	✓	✓
Regional care setting	✓	✓	✓	✓	**x**	✓
Remote care setting	✓	**x**	✓	✓	✓	✓
Health system settings (x 5 jurisdictions)	✓	✓	**x**	✓	**x**	✓

1 = see text for details of treatment types; 2 = first language is English, first language is not English ✓ = achieved x = not achieved;

Including non-Indigenous patients provided us a comparison group. Equally importantly, it sharpened our understanding of what culturally-based differences might involve for Indigenous patients and how they might play out to differentially influence outcomes. We applied the same guidelines in recruiting all patients (e.g. range of treatment types, equal numbers of males and females etc) including, where possible, patients who spoke English as a second-language.

The contingencies of workplace-based research meant applying the patient inclusion characteristics as 'guidelines' rather than rigid 'criteria'. Aspects of service sites – especially size, but also staff workloads, the health status of individual patients at the time, the events of the particular day, the availability of off-site patients, and, to some extent, the level of key staff interest in the work of IMPAKT – all influenced the possibilities and process of recruitment (see Research settings). (Although we use the term 'workplace' to conveniently convey a distinctive aspect of the research, the 'workplace' of staff is also the 'treatment centre' for patients).

Achieving the diversity necessary to explore the IMPAKT questions at a national level required a much larger than usual number of interviews for this research methodology. For example, the 146 Indigenous people interviewed were receiving their treatments at 21 different service centres – so there were not necessarily large numbers of patients per site. On the other hand, those particular 21 sites account for a significant majority of the total number of Indigenous patients.

#### Staff and nephrologists

We recruited staff primarily on the basis of their day-to-day decision-making roles in relation to:

• Indigenous patients accessing transplant; and

• managing and providing dialysis services.

These individuals, especially nephrologists, not only had extensive experience with patients, they had insights into local service arrangements, had intimate knowledge of crucial decision-making points and were themselves highly influential, if not responsible, for existing patterns of transplant access. We also sought senior renal nursing staff who were (clinical) managers of service centres.

A second basis for staff recruitment was the level of involvement in specific processes identified in the Steps model, namely:

• the process of educating and informing patients;

• co-ordinating the transplant 'work-up' processes; and

• involvement with patients in decisions concerning treatment management.

Renal units had varying configurations of renal nursing staff to cover these roles. For example, some, but not all, centres had a dedicated (nurse) transplant co-ordination position; most had a dedicated patient education position though not all allocated a full-time position; some centres had Aboriginal Health Workers, most did not; renal technicians were rare and so on. At each service centre, we sought out the full range of personnel who might be expected to be involved in these processes, including non-clinical staff, particularly social workers and support staff.

Third, in keeping with our objective to maximise involvement of Indigenous people, we made special efforts to invite Indigenous staff working with renal patients to participate in the Interview study. With one notable exception, Indigenous staff did not hold key decision-making positions, and on the whole were clustered in the roles of Aboriginal Liaison Officer or Aboriginal Health Worker.

### Engaging colleagues and communities

From the outset, we sought to build participatory relationships with service providers. This process began before the funding application was submitted in 2002, through discussions with nephrologists working at various transplant units and regional hospitals, as well as with community-based primary health care agencies controlled and managed by local Indigenous communities. One or more nephrologists from each of the eight main participating hospitals became an associate investigator for the IMPAKT study.

Following the start of the research program in early 2004, and the appointment of an Indigenous health researcher (CP), IMPAKT began to systematically engage relevant Aboriginal and Torres Strait Islander communities and organisations. Similarly, the team nephrologist/researcher (AC) managed relationships with the nephrological community. Although not rigid or prescriptive, this division of responsibility helped IMPAKT grasp issues of concern to our principal stakeholders. The original funding application was supported by a letter from the Northern Territory chapter of the National Aboriginal Community Controlled Health Organisations, an umbrella group for the Aboriginal community controlled health services (ACCHO). Although such services provide comprehensive primary health care, with one notable exception they have limited clinical involvement in renal replacement therapies. However, they are the key service providers in the early stages of care of people with kidney disease.

Early in the project, IMPAKT wrote to all local ACCHOs in the region of the eight participating hospital sites, informing them of the study and inviting their comment on the proposed research. This usually led to a personal contact with the Indigenous researcher and a one-on-one explanation of the IMPAKT program. In other cases, the Indigenous researcher followed up the introductory letter with phone and email contact.

A round of introductory presentations at ten of the participating service sites, with an additional two sites linked in by video, preceded fieldwork. Attendees included key hospital renal and other staff, nephrologists and invited local ACCHO staff. These audiences responded critically and reflectively on a wide range of issues of local concern. Subjects raised were as varied as the validity of assumptions underlying the model (e.g.*Is *transplant the optimal treatment?), the value of the research (e.g. Indigenous people would be better served by concentrating on *preventing *kidney disease.), the methodology (e.g. What about patients that don't speak English?), the probabilities of success (e.g. 'Our' patients are unlikely to talk to you.) and so on. These conversations added greatly to the next iteration of the project design.

At each introductory presentation, IMPAKT invited staff to establish a local 'reference' group [see Additional file 1]. On our return for the field work period, the local reference group advised on 'ground rules' for the site-based activities, including suitable times/places for interactions, appropriate processes for managing (potential) issues or complaints arising from interviewees, and accessibility of counselling services. IMPAKT had formal institutional ethics approvals in place, however, the site-based reference groups provided a point of contact for feedback to the research team and represented staff and institutional interests during the course of the research.

In several, but not all sites, ACCHO staff participated in the site-based reference groups. IMPAKT gave formal introductory presentations at four ACCHOs and made contact with the local ACCHO at every site. There is currently no agreed basis for collaborative involvement in Indigenous ESKD patient care between the state renal treatment services and ACCHO agencies. With one exception, ACCHOs generally have little routine contact with the dialysis treatment centres although they often provide services to individual ESKD patients. All expressed strong interest in the issues affecting patients and emphasised the need for preventive health measures to reduce the levels of kidney disease.

The timing of field work at each site was worked out in consultation with local associate investigators and senior relevant nursing staff. As the field work period drew near for a particular site, IMPAKT contacted the associate investigator(s) at the site as well as the relevant senior nursing staff. We sent a number of locally-badged small posters to be placed in the unit inviting patients to participate in the study, providing dates of the visit and photos of the interviewers [see Additional file 2]. We had a logo prepared for use on all correspondence, and the IMPAKT interviewers wore identical T-shirts carrying this logo. This simple level of identification was highly effective, especially in institutional settings. It particularly assisted patients to identify us as we worked through a multi-step informed consent process.

Throughout the project, IMPAKT produced a 2 to 4 page quarterly newsletter [see Additional file 3]. In accessible, non-technical language and with a balance of visuals and text, it targeted patients as much as staff – especially Indigenous patients. The newsletter, distributed both electronically and in hard copy, maintained continuity between the participating sites and IMPAKT and also disseminated information about the research more broadly.

### The research team

IMPAKT benefited immensely from the multi-disciplinary skill base of its core working group which included:

• A nephrologist/researcher (AC) who has combined clinical training and work experience with Aboriginal renal patients, with training in public health and health services research;

• a social epidemiologist (JC) with experience in social determinants of health and health services research, particularly in relation to Indigenous Australians;

• an anthropologist (JD) with lengthy Aboriginal community experience and research interests in health, health services and communication;

• an Indigenous researcher (CP) with a background in health and experience in Aboriginal Community Controlled Health Services; and

• a PhD candidate (KA) with a background in health psychology, who is being mentored by the research team to develop her skills in qualitative and Indigenous health research.

From time to time a further three clinical nephrologists were also involved. One (PS) had extensive experience in leading a clinical and state-wide service providing care to a predominantly Aboriginal clientele across urban, rural and remote communities. A second (JE) is a leading transplant nephrologist with key responsibilities in the development of national policies regarding kidney transplantation. The third (MJ) is a nephrologist who recently completed training and is undertaking post-graduate study.

Team members lived in Sydney, Cairns and Darwin. The overall program management fell to AC and JC, however, relations within the team were distinctly egalitarian. Planning was a continuing, collaborative activity through weekly teleconferences. A crucial early appointment to the IMPAKT team was an Indigenous health researcher (CP) who took a leadership role in relation to Indigenous issues and managed the Indigenous community engagement aspects of the Interview study. Her previous research experience in kidney health and her clinical experience as an Aboriginal Health Worker proved invaluable. Equally, CP was a post-graduate student and benefited from being part of a multi-disciplinary team working on a national research program. Our team also included an (initially) part-time team member (KA) who provided critical logistical and data management support to the interview team during and following the field work period. Gaining a postgraduate scholarship during the early phase of IMPAKT, KA subsequently took up a specific aspect of the emerging IMPAKT data as the focus of her PhD studies under the supervision of team members (AC, JC). For a five week period, she joined the interviewing team (JD, CP) to gain experience of community-based fieldwork and working with Indigenous participants. She was mentored in this by both JD and CP.

#### Managing the study

Weekly telephone conference meetings, the cornerstone of IMPAKT project management were operational in nature and minutes were taken. During a lengthy field work program we maintained phone and email contact and were able to problem solve collaboratively 'on-the-spot'. One team member (KA) provided dedicated logistical field support from the George Institute. At strategic points in the program – pre-field work, pre-data collation, pre-analysis – we held face-to-face planning and reflection meetings. During these meetings the Steps model and our initial research questions were revisited in light of accumulating data – not only field data, but emerging literature, conferences attended and so on. Team members acquired training in specific skills including the three principal interviewers undertaking a three-day intensive training course in QSR NVIVO, our preferred data management software.

### Ethical review

The IMPAKT Interview study was approved by 14 separate ethics committees, each with different application requirements. The committees included the participating hospital sites with their hospital/university committees, the various state-based health departmental committees and six Indigenous-membership-only committees. We spent three months of 2004 submitting applications and responding to queries. Some decisions took several months to finalise. In some cases hospital/university committees, on learning that the project had also been submitted to a specific Indigenous research ethics committee, delayed their own decision until that of the Indigenous committee came through. This process would benefit from streamlining, but the 'wait and see' responses of several large institutional ethics committees raises questions about their confidence in their own ability to review research with Indigenous people.

Ethical approaches to research with Aboriginal and Torres Strait Islander peoples requires attention to a wider range of matters than are usually addressed through institutional ethics approval processes [36]. As we were working with a minority group likely to have a level of distrust of signing official documentation [37], our process of ensuring informed consent needed considerable thought. In addition we aimed to include people who were non-English speaking and poorly literate. Setting up reference groups at each participating site and the involvement of local ACCHO were measures to ensure the well-being of all local participants, but especially the patients. In all cases participation was voluntary. Under Australian ethical guidelines researchers may not offer incentive payments to participants, however IMPAKT provided a light lunch at all site-based staff meetings. Similarly we provided small gifts of food to patient participants and renal units following speaking with them.

#### Informed Consent Materials

Consent materials comprised a Project Information Sheet and an Informed Consent Form. We made particular effort to use clear, accessible language in these forms. We produced a 'professional' as well as a 'patient version' of the Project Information Sheet [See Additional file 4; Additional file 5]. These forms were site-specific, included appropriate logos and the names of local associate investigators. All participants used a common version of the Informed Consent Form [See Additional file 6]. It covered the interview itself including format (recording or notes) as well as seeking permission to use interviewees' words in future publications. It also covered interest in receiving a transcript of the interview and sought instruction on dealing with the audio record at the end of the project. There is a legal requirement for research data to be archived for a set period, but our question specifically addressed cultural issues associated with holding and preserving the audio records of a person who may have subsequently died. In some areas, Indigenous people prefer not to preserve the 'voice' of a deceased person. A total of 125 patients, including 76 Indigenous people requested their audio interview be destroyed. Participants were given a copy of their signed consent form at the time of the interview.

### Kinds of data

Five different kinds of data were collected at most locations including:

• Interviews of key groups – patients, nursing staff and nephrologists;

• A description, from the viewpoint of health professionals, of a kidney patient's journey – a focus group mapping exercise conducted at 13 service centres;

• An evaluative description of kidney patient education processes and their supporting educational resources;

• Ethnographic observations at dialysis service centres; and

• An assessment of Indigenous patient knowledge concerning transplant.

Different interview schedules were prepared for each of the following categories of participants: patients, nephrologists, renal nursing staff, renal patient educators and renal social support staff. The final interview formats varied from structured questionnaire-type, to semi-structured and open-ended, with a further set based on a narrative format. Most were conducted face-to-face and recorded as audio files; 14 were telephone-based (and recorded).

#### Common thematic core

While designed to account for a range of roles, the interviews addressed a common set of themes relating to the main questions of the Interview study:

1) information and communication processes;

2) decision-making and treatment options;

3) attitudes and views on transplant;

4) views on 'compliance'; and

5) the local system and its context.

#### Nursing and other staff interviews

The initial interview schedules were developed through multiple drafts and prepared in relatively structured formats [see Additional file 7; Additional file 8]. The interview schedules were piloted with one or two individuals and further refined; they had a planned duration of between 45-60 minutes. Aware of time constraints in workplace-based interviews, the team debated the relative value of inclusions or exclusions. At the time of interview however, each interviewer determined whether the exact wording – or even the question – was put. Interviewers regularly sacrificed the prepared 'structure' – though not the themes – in pursuit of meaningful engagement. Questions were adapted to specific circumstances and interviewers were responsive to interviewees' comments.

Potential interviewees were given a brief description of the themes or topics of interest. Interviewers attempted to establish a 'conversational' rather than 'interview' atmosphere and used the question sheet more as a reminder list than a script. While the question schedule was not in any way concealed, neither was it provided unless requested. The language used (in staff interviews) was generally that of service providers, and terms such as 'compliance', for example, were presented initially as unproblematic to draw out respondents' views.

In addition, staff were invited to rank, on a 1–5 scale, the extent to which their workplace emphasised the following: efficiency, economy, patient-centred care, shared decision-making, clinical excellence, staff development and patient development/empowerment.

#### Nephrologist interviews

The nephrologist interview schedule was prepared through a similar process as other staff interviews and piloted with two nephrologists. The planned duration of an interview was between 45–60 minutes. The interviewers were fellow nephrologists and interviews were undertaken face-to-face by AC and MJ during breaks at scientific meetings and elsewhere. Thus nearly all interviews happened away from the usual work-site.

As with the interviews of nursing and other staff, a consistent set of themes was explored in each interview and interviewees were given a brief description of the themes or topics of interest. Again, however, the wording of questions, order of questions and structure of the interview, was frequently changed in pursuit of meaningful engagement. Nephrologists were also invited to rank, on a 1–5 scale, the extent to which their workplace emphasised: efficiency, economy, patient -centred care, shared decision-making, clinical excellence, staff development and patient development/empowerment. [See Additional file 9]

#### Patient interviews

The challenges of involving Indigenous people in health research are well attested [38-40]. In the case of IMPAKT we were seeking to involve Indigenous people suffering a life-threatening chronic illness, many of whom were both non-English first language speakers and remote community residents. Many of that target group additionally have poor literacy and little or no comparable previous experience of conversations constructed primarily as research tools [41]. Such linguistically and culturally different groups present significant challenges to 'standard' research methods both in attaining validity [42-45] as well as addressing concerns of ethical practice [46]. So, while we included both Indigenous and non-Indigenous patients, the IMPAKT patient interviews were designed *primarily *to facilitate Indigenous participation, on the assumption that such an approach would present no barrier to the participation of non-Indigenous people.

The patient 'interview' structure was moved towards that of a life story narrative with coherence from the *patient's point of view *[45]. The life story narrative created a conversational frame that was both recognisable as well as engaging for patients. We reserved the most delicate questions (concerning attitudes to transplant and related matters) to the latter stages of the interview when a measure of rapport had been established.

While the patient interview schedule incorporated the five common themes, they were approached differently through inviting the patient to describe the sequence of what had happened to them, how it had affected them, and their understanding of their options. From the interviewers' viewpoint each interview included the following themes: personal health history; social and psychosocial context; attitudes/values, treatments, information and communication, transplant and satisfaction with services.

Three versions of the patient interview schedule were prepared [see Additional file 10 Additional file 11; Additional file 12]. First, a version that an interviewer might use; second, a version that was pared back to a series of interviewer prompts; and third, a highly compressed, user-friendly version, that could be shared with patients and their families – as a response to the anticipated, slightly apprehensive: *What do you mean questions, what sort of questions? *Although this latter version was minimalist, it was by no means accessible to many of those we subsequently interviewed.

#### Peer interviewing

Peer interviewing was a feature of the IMPAKT approach. Team members with specific experience and knowledge had a leading – though not exclusive role – in interviewing participants with similar interests and experience. The IMPAKT Indigenous team member led the strategy of IMPAKT's engagement with Indigenous organisations and individuals. The high number of Indigenous patient interviews precluded her doing them single-handedly and both non-Indigenous team members, one of whom had considerable relevant experience (JD), also interviewed Indigenous patients. Similarly the nephrologists on the team managed the IMPAKT interactions with the nephrologists. Sharing the specialised content knowledge of kidney disease and transplant was thought to produce a more insightful discussion of a complex decision-making process. It was also thought that a professional colleague as interviewer would be accorded more attention. The peer interviewing approach undoubtedly underpinned IMPAKT's success in recruiting Indigenous people and specialist clinicians – both being groups that could be described as 'hard-to-reach' in different respects.

### Research settings: dialysis treatments and service centres

Dialysis and kidney transplant are the two basic categories of treatment for end-stage kidney disease. Dialysis involves removing wastes from the blood across a membrane. This is done either externally using a dialysis machine, termed 'haemodialysis', or internally across the peritoneum (membrane lining the gut) which is termed 'peritoneal dialysis' [see Additional file 13]. Patients may move between these various kinds of treatments during the course of the illness. Both peritoneal and haemodialysis can be done at home by the patient. However, IMPAKT patient interviewees were predominantly, but by no means all, on haemodialysis treatment in dialysis service centres where nursing staff were in attendance. From the research interviewers' point of view, it was more difficult to meet people on any of the home-based treatments, since our consent process required staff to actually phone or email individuals about the project rather than speak with them during their three times weekly dialysis sessions. Moreover, there were limits on the time we could allocate to setting up interviews as well as the distances that we could travel to interview people in their homes during our field visits.

#### Dialysis Service Centres

The majority of the IMPAKT interviews took place in dialysis service centres. Centres treating the most unwell patients with complex or acute conditions are located within larger hospitals ('in-centre'); other patients receive dialysis treatment in stand- alone service centres ('satellite units') often, but not always, co-located with hospitals (see Additional file 14; Additional file 15; Additional file 16]. IMPAKT did not recruit participants in large hospital 'in-centre' units.

A dialysis service centre comprises a series of patient dialysis points arranged around a room. Each dialysis point consists of a single large recliner-type arm chair with independently moveable sections for elevating either the patient's legs, or upper body. The chair is covered by a cotton sheet, has a light hospital blanket and one or more pillows. Beside each chair stands a mobile, 1.5 metre high, box-shaped computerised dialysis machine. Each point also has a mobile tray and a rubbish bin [see Additional file 17]. Preparation for each patient's 3–6 hour session of dialysis involves the dialysis staff – sometimes the patients themselves – setting up the dialysis machine, attaching the multiple sections of disposable plastic hosing, the plastic dialyser, and the various solutions and lines that are part of the treatment process. Each patient is then connected into a dialysis machine. Connection entails placing two needles into a specially prepared entry point into the bloodstream, usually on the lower forearm [see Additional file 18]. Barring problems, the patient then remains in the chair, connected to the dialysis machine for varying periods of between 3 and 6 hours. Patients spend this time variously, reading, watching television, sleeping, listening to music etc. The insertion and removal of the two needles marks the beginning and end of each dialysis session for each patient. These are the busiest periods in the session as patients arrive and leave and available staff move from patient to patient disconnecting some, and connecting others.

The chairs – numbering between five and eight for the smaller service centres and upwards of 25 for larger ones – are arranged in various configurations along the walls (see Additional file 19]. All centres also had wall and/or ceiling mounted televisions. Most – but not all – provide patients with a light snack and tea or coffee during their session. Air conditioning tends to be set at very low temperatures; certainly Aboriginal patients in the tropical northern regions find the climate overly cold and use additional covers. Service-centres ranged from purpose built, well-designed, 'patient-friendly' facilities to a small number that were cramped, aged and decidedly 'make-do' where patients and staff worked around the obvious difficulties.

Haemodialysis is not a benign treatment. While many sessions are uneventful, individual patients regularly experience severe cramping and discomfort, others have sudden blood pressure variations and, in the worst cases, heart attacks and death. Some sites did not provide cubicle curtains; patients then had both very limited privacy and distressing exposure to emergencies affecting other patients. Indigenous patients in particular often showed signs of debilitating levels of tiredness as they came off the dialysis machines. Outside the fifteen or so hours spent on the dialysis machine each week, patients are also under permanent severe fluid intake restrictions, pervasive dietary regulation and, for the majority, complex medication regimes.

#### Centres and settings

The service centres were in a diverse range of geographic localities and settings (Figure 2). On a national index of remoteness (Accessibility/Remoteness Index of Australia, ARIA) that ranks particular localities by a combined consideration of distance and access to services including health, transport and education, the participating dialysis service centres were in localities ranging from the 'highly accessible' (HA) to the 'very remote' (VR) – such as places in the Northern Territory, or the north of Western Australia (Table 1, Figure 2). The degree of remoteness is likely to affect the convenience of coordinating inter-sectoral, inter-department, or inter-organisational arrangements. For example, arranging a specialist's appointment in Perth for a self-care dialysis patient living in a community ranked as either Remote (R) or Very Remote (VR) is a complex and costly exercise, involving several people who organise flights, accommodation, city transport, paperwork and so on in addition to supporting the patient to understand and carry through the arrangements. Indeed, in the context of Australian geography and population distributions, the same is true even for places ranked as Moderately Accessible (MA). Around 70% of the interviewees, both patients and staff, but not kidney specialists, were living and working *outside *areas considered 'highly accessible'.

### Fieldwork at dialysis service centres

The patient/staff interview team usually comprised two travelling interviewers (JD, CP) with a third person (KA), based at the George Institute for International Health, providing fieldwork support and managing incoming data, including the review and return of transcripts to participants. However, all three team members (JD, CP, KA) carried out interviews in the two most populous states. The fieldwork was continuous during a 12 month period (early 2005–2006) and included, for members of the patient/staff interview team, 30 weeks of time away from home. The nephrologist interview team comprising two nephrologists (AC, MJ) based at the George Institute did not work in dialysis service centres and organised their interviews separately.

#### Recruiting patients

As the period of field-work at a particular site drew near, we contacted local site Associate-Investigators as well as those who had volunteered for the local site Reference Group. Providing an outline of the patient recruitment guidelines, we asked them to begin identifying potential patient candidates.

Patient recruitment on site followed several stages (Table 3). The phased or 'segmented' form of recruitment aimed to minimise perceptions of applying pressure on patients to participate, since many were essentially 'captive' while on haemodialysis in the service centre. Indigenous people have made clear their preference for longer timeframes as well as less individualistic, unilateral decision-making processes concerning research participation [47]. IMPAKT interviewers encouraged patients to speak with other members of their family about the project, to show them the project information sheet and to discuss the worth of the project with others before making a decision.

**Table 3:** Sequence of recruitment and informed consent process

**Stage**	**When**	**Activity**
1	3 weeks pre-field work	send recruitment guidelines; staff begin identifying potential participants
2	on site	staff enquire if patient is interested in hearing about study
3	on-site	staff introduce IMPAKT interviewer to interested person, or provides patient contact details
4	on-site	interviewer explains project to patient, provides patient information sheet
5	on-site	interviewer re-visits patient, if willing to participate, they nominate interview time
6	on-site	interviewer meets and speaks with patient, completes informed consent paperwork
7	home base	send transcript of interview to participant, including letter of thanks; 6 weeks to amend

First, a staff member made an initial enquiry as to whether individual patients were interested to hear about the IMPAKT proposal. If they agreed, and were satellite dialysis patients, the staff member introduced them to one of the IMPAKT interviewers. If they were transplant recipients or doing their treatment at home, the staff member sought permission to pass on their contact details to an IMPAKT interviewer. Second, the IMPAKT interviewer explained the nature and purpose of the Interview study, answered questions, explained the consent format and provided them with a project information sheet. The patient was invited to participate and the interviewer arranged to contact them the following day or so to ascertain their decision. If agreeing to participate, the patient nominated a time and place for an interview. At the time of interview, the informed consent materials were re-explained and signed. As far as contacting 'off-site' patients – transplant recipients and people doing their treatments at home – we were, of necessity, guided by the suggestions of the local staff.

#### Recruiting nephrologists

At each participating site, where a substantial number of Indigenous patients were receiving renal care, AC directly approached the head of the renal unit to become an associate investigator on the IMPAKT research program. In addition, each of these investigators, and other nephrologists at each site, participated in the Interview study. The interviews were conducted either face-to-face at professional conferences and gatherings or by phone.

Interviews were carried out on a 'convenience' schedule and their timing was unrelated to the concurrent program of patient/staff interviews. We provided project information sheets (the professional version) and an outline of the specific topics for discussion with nephrologist participants. All of the nephrologists approached agreed to participate in the interview study.

#### Recruiting staff

Field work at each site began with a meeting with the local site reference group (see above) many of whom were renal nursing staff. At this meeting, staff were invited to be participants in IMPAKT. They also identified other key non-nursing renal staff such as social workers and Aboriginal liaison officers. We provided project information sheets (the professional version) and an outline of the specific topics for discussion with staff participants. Since we were several days or longer at each site, we met most senior renal nursing staff and, depending on the size of the service centre, many of the other staff. As opportunities arose, we explained the IMPAKT project to individual staff and invited them to participate. As with patients, staff recruitment was a staged process with time between the explanation, the decision to participate and the actual interview. Workplace factors (time, workload, and daily happenings) determined whether or not interested individuals ultimately participated. In only a few instances, key staff were unable, or chose not to participate.

#### Interviewing in a dialysis service centre

It was possible to move around a dialysis service centre (satellite unit) and speak with individual patients once most had settled for the particular session. Sitting beside the patient, between neighbouring machines, we conducted interviews using a very small, inconspicuous digital recorder and a small lapel microphone (see below). Dialysis units, especially the larger ones, can be noisy places with machine alarms regularly going off, with nurses coming and going and with multiple televisions/videos playing. To some extent this created a noise 'curtain' to give some privacy to patients who chose to speak with us.

Service centre size, measured by number of chairs, was a key attribute determining likely participation of patients in the Interview study. Dialysis service-centres with between 5 and 10 chairs were characterised by a higher degree of familiarity between patients and staff, as well as between patients themselves, than was seen in larger service-centres, where chairs numbered upwards of 20 and more. The latter were large and busy spaces where people were almost continually being either put on or alternatively taken off their dialysis machines. In contrast, in the small service-centres, the patients were soon settled and a period of relative quiet ensued.

The IMPAKT interviewers, as 'strangers' wearing distinctive clothing and sitting down talking to individuals in the smaller service-centres, were highly visible. This generated some interest among other patients, particularly in service-centres with predominantly Aboriginal patients, as to what we were doing. Questions about the project led to further distributions of the Project Information Sheet, often then to an offer of opinion and an interview. Since we worked at each service-centre for up to two weeks, there was time for people to observe us as well as to hear from other patients and staff what we were talking about. The measured pace and lack of pressure to participate were important dimensions of our approach. The patients chose the location for the interview; with few exceptions people chose to speak during their subsequent dialysis session – it was convenient for many, and passed the time for some. Indigenous patients however were highly likely to sleep during dialysis and our arrivals needed to be well timed.

Dialysis nursing staff interviews were fitted in at the convenience of the staff and the service centre work schedule. Most were completed in a tea-room, an office or other meeting space. Non-nursing staff (social workers, Liaison Officers etc) were generally spoken to in their offices.

#### Documentation of interviews

The documentation for each interview comprised a signed consent form, an audio file, a 'Record of Interview' form, a transcript that had been checked against the audio and, where requested, confirmed by the interviewee.

The 'Record of Interview' form included additional information including date, place, interviewer and socio-demographic data on interviewees. Three versions (renal nursing staff, educators, patients) of the Record of Interview form recorded data relevant to the respective groups as well as interviewees' opinions on the quality of service they were either receiving or providing (see Additional file 20; Additional file 21; Additional file 22].

#### Recording and transcribing

We recorded interviews with small (10 cm × 4 cm) Sony digital recorders (ICD MS525) used with external lapel microphones (Sony ECM-T6/ECM T-8). At the end of each interviewing day, interviewers uploaded the audio files onto a laptop and backed them up on memory sticks. Audio files are very large in size – typically an interview was between 5–10 MB in the highly compressed file format of the Sony recorders. More problematically, when converted to one of the more common standard formats (e.g. WAV) they increased in size by a factor of 10. This was a constraint in moving the audio files around electronically. Fieldwork sites ranged from cities to remote locations. Where possible, audio files were uploaded to a password-protected web folder of the home base (The George Institute), otherwise they were loaded onto memory sticks and posted back. Once at The George Institute, the audio files were uploaded via a password protected website to a commercial transcribing service. Uploading of audio files and downloading of draft transcripts was secure and password protected. Additionally the work was covered by a contract between IMPAKT and the transcribing company concerning the confidentiality of the material and its destruction following the return of completed transcripts. The transcription service was competitively priced, good quality and provided fast (standard 7 days) turnaround. Transcripts used a standard template in Word format. The cost per transcript (audio time around 45–60 minutes) averaged around $70.

Each interview draft transcript was returned to the original interviewer for checking against the audio. This was assisted by use of a USB-type transcribing foot pedal (Olympus DSS Player Pro with RS24 Foot Switch). After corrections, the transcript was returned to those participants who had so requested at the time of interview. A covering letter invited them to amend and return the transcript within 6 weeks. With one exception, no participants sought to amend the transcript of their interview. In one case a participant sought to withdraw the complete interview, expressing concerns about anonymity and job security in a politicised local service environment. After discussion, the participant consented to retain most of the original interview and to re-interview on a few specific questions.

### Profiles of interview participants

A detailed description of the characteristics of the interviewee participant groups will accompany specific outcome publications. For a summary description see Table 1 and Table 4.

**Table 4:** Roles and positions of staff interviewees*

**Position/role**	**Non-Indigenous**	**Indigenous**
Nephrologist	19	0
Renal Nursing Staff	67	3
Renal Nurse NUM/CNC	20	0
Renal Nurse/Patient educator	11	0
Renal Nurse/Tx co-ordinator	7	0
Renal Technician	1	0
Aboriginal Health Workers	0	5
Social Workers	9	0
Indigenous Liaison	0	9
Other	5	1

* Renal nursing staff (67 in total) may have more than one role and may therefore be represented in this table more than once

### Communication issues

IMPAKT's challenge was to engage Indigenous patients – members of a 'hard-to-reach' and vulnerable population. One significant dimension of the 'hard-to-reach' label arises from Indigenous Australians' shared historical experience of colonisation and the on-going consequences, including health consequences, of decades of social and economic marginalisation. Officials, administrators, police, medical personnel and now researchers may be sceptically received as "more of the same", a view Indigenous people may have in common with other contemporary minority populations [39,48].

A second dimension of the 'hard-to-reach' description arises from the linguistic, social, and cultural differences as well as the varied historical experiences of contemporary Indigenous Australia. Approximately 74% of Indigenous Australians live in urban and/or regional Australia; the remaining 26% live in remote/very remote areas where they often comprise a majority of the local area population [1]. Those from the more remote areas are more likely to be speakers of an Indigenous language as their first language and to hold views of illness and wellness that are strongly influenced by traditional practices. They are *less *likely to have had experience of large hospitals and institutionalised care, urban living and formal schooling beyond primary level.

In areas where Indigenous languages are spoken, there are a multitude of different languages, few of which are mutually intelligible. Of the 50 or so languages which remain (from an estimated pre-colonial 200 or more) only two now have more than 3,000 speakers [49] and none have more than 5,000. Interpreters are not available for many language groups and even where they are, the interpreting process may still be problematic (see below).

Historically, Indigenous Australians had an oral cultural tradition. Languages were not written. Subsequently, Indigenous people have had limited access to formal education and contemporary education levels, especially among remote living communities, remain comparatively low [1]. However, regardless of where they currently live (urban or remote), Indigenous participants emphasised the importance of communicating in formats other than text alone.

For those who speak English as a second language however, perhaps of greater significance than the language difference itself is the lack of shared conceptual and/or cultural knowledge. Translation does not necessarily address this. From the IMPAKT perspective this had two important consequences. First, we (as interviewers and interviewees) did not necessarily *share *a conceptual framework within which to discuss ESKD and transplant (how illness is caused; how wellness is achieved; where transplant kidneys come from, etc). This gap in understanding similarly challenges the health system [18]. Second, it was equally uncertain as to whether we shared an understanding of the underlying *purpose *of the interview and associated activities – what 'research' is, what sort of 'knowledge' might be acquired in this manner etc. Linguistic, literacy, conceptual, cultural and experiential differences converge in this situation to seriously challenge conventional health research methods in terms of both validity as well as logistics. This is not to suggest that communication is therefore impossible, or that these kinds of differences are only experienced by Indigenous people. However, it is this same bundle of characteristics that profoundly complicates the process of negotiating the alien world of a large, English-language dominated, Australian hospital system.

The situation proved a considerable challenge for IMPAKT especially in the Northern Territory -where the overwhelming majority (90%) of patients are Indigenous and have come to treatment from remote/very remote communities. To illustrate: working in Western Australia in metropolitan, regional and remote areas, three IMPAKT interviewers completed 98 interviews including 45 Indigenous patients in 13.5 person/weeks. In the Northern Territory, by contrast, it took 52 person/weeks to complete 55 interviews including 43 Indigenous patients. The task was only practicable because one member of the research team (JD) lived in Darwin and was therefore able to work on the Northern Territory interviews concurrently with other activities at minimal marginal cost.

#### Interpreter use

It was our intention to use interpreters as needed, however we did few interpreted interviews during fieldwork visits. Only two service centres regularly (though not necessarily routinely) used Indigenous interpreters. Other jurisdictions suggested that Indigenous patients did not require interpreters and the service was unavailable. Of the non-Indigenous interviewees, only one required an interpreter. The issues involved are complex and difficult to address, particularly where speaker communities are relatively small [50]. However, to deal with some of the issues outlined above, IMPAKT then made a particular effort to undertake a set of interpreted interviews with Indigenous people. It not only offered us a comparative view on the English language interviews, but gave us a more nuanced account of patients' perceptions and attitudes to treatments. IMPAKT sub-contracted out seven interviews to two locally-based non-Indigenous people who are fluent speakers of Pitjantjatjarra, an Indigenous language of Central Australia. The contract-interviewers were briefed about the project and provided with a package containing the project information sheets, informed consent forms and all three versions of the patient interview schedule. Each of the completed interviews was around 30–40 minutes duration.

The usual sequence of transcription of the spoken Pitjantjatjarra into a written form, then translation of that written version into English was prohibitively time-consuming and expensive. Instead an IMPAKT interviewer (JD) familiar with the languages of the area, together with each contract-interviewer, worked through the interviews. Set up with another recorder, the non-Indigenous contract-interviewer interpreted directly into English both their own original questions and the participant's response. This audio version (all in English) was then transcribed by the project transcribing service. These transcripts however required substantial further work by the IMPAKT interviewer. The process was a critical exercise in validation of other (non-interpreted) interviews but each interview was both lengthy and expensive at around $1000 per interview.

#### Distress and emotion in interviews

Recounting one's experiences and problems living with ESKD can itself be a stressful exercise. All three interviewers experienced some patients becoming emotionally upset during their conversations. Although on each occasion an offer was made to discontinue the interview, this was not taken up. The participants regained their composure and continued; indeed many patients remarked on the therapeutic value of recounting their personal experiences. Among staff also there was a small number who showed a level of distress mainly related to the difficulties of Indigenous cases and the unrelenting workloads associated with their present workplaces.

### Managing and analysing data

Interview transcriptions and information on the Record of Interview Sheets, including socio-demographic data on participants, responses to ranking questions, the answers to the Education interview schedule and the field diaries have been entered as a project in NVIVO (QSR, NVIVO7, Doncaster Australia), a program for managing and analysing qualitative data. The process maps and the collection of educational resources are held separately from the NVIVO project.

Although the IMPAKT interviews constitute the primary data for analysis and interpretation, they have been part of an on-going analytic process. The weekly meetings were the main forum through which this happened. Site visits and individual interview experiences were discussed between the interviewers in the field and then collectively with the team during the regular meetings. Concurrent with the interview field work, team members (AC, KA, JD) have given conference presentations and other public talks presenting 'emerging' themes and preliminary data. Audience responses and discussion were fed back into our team discussions. Papers on the completed, related sub-studies were also prepared during this period. Going back and forth between, on the one hand, the particularities of interview data and field observations, and, on the other, the Steps model, our key questions and the research literature, provides the foundation for the interpretations of the Interview study data. This kind of research 'tacking' has occurred through the life of the study. While there was (and is) a large amount of crucial interpretive analysis to do, specifically on the interviews themselves, it remains one component of a broader, continuing process involving the IMPAKT team.

#### Coding: Themes and sub-themes

NVIVO software enables multi-level coding of text. 'Coding' is the process of allocating interviewees' commentary, or any 'text' (including whole documents, photographs etc) against a set of identified analytical categories ('nodes' in NVIVO terminology) such as themes and sub-themes, topics of interest or similar. The NVIVO program is highly flexible. It allows nodes to be individually defined, it supports the allocation of text to multiple nodes, as well as the capacity to add, delete, merge, re-define and otherwise change nodes at any point during the coding process. Searching and context restoration (seeing particular sections of text in their original context) are also useful features; nodes can be tracked quantitatively as well.

The IMPAKT coding process involved several preparatory steps:

a) A select set of interview transcripts (11) were read by the team; major themes and points of interest were identified and discussed, and the purpose and priorities for coding reviewed.

b) Using NVIVO, interviewers (JD, KA) independently coded four of these interviews using the original five common topics as a basic framework and identifying further themes/sub-themes.

c) The two resulting coding reports were compared and through a process of deleting, merging and re-definition, a consolidated 'coding dictionary' of nodes was produced.

d) On the basis of interest and time available, the node dictionary was split between the two coders. (These two coded databases were ultimately merged.)

Each interviewer then coded *every *interview against their set of nodes. The coding process has both a deductive dimension (with the five common topics as an organising framework) and an inductive (ground-up) aspect, where additional themes and sub-themes emerged directly from the data [51] and see Table 5. So, both interviewers added new nodes. Prior to merging the two sets of coded interviews, the accumulated new nodes were rationalised. As with the first round of coding comparison, this rationalisation involved a detailed discussion of each interviewer's additional nodes, comparing definitions, checking against the actual interview selections, merging similar nodes and determining the best location for nodes. This resulted in a finalised, consolidated code dictionary for future use with the data base.

**Table 5:** Examples of coding types from IMPAKT (after [51])

**Type of code**	**Examples from IMPAKT (theme level only)**
Conceptual codes	communication; transplantation; education; 'compliance';
Relationship codes	relationships (staff, patients); broader management issues;
Participant perspectives	psycho-social issues; knowledge & understanding; education; emotional states; compliance; transplantation; broader social context;
Participant characteristics	socio-demographic information; emotional states; staff worldviews
Setting codes	renal system and organisation; broader management issues; suggestions; geography

The NVIVO program tends *not *be used in a multi-user environment and anticipating the final merging process was somewhat nerve wracking, although in the event, it was trouble free. Nevertheless considerable vigilance was required during the period of coding to keep the two (as yet unmerged) versions identical, apart from their respective coding patterns. Failure to achieve this would have substantially increased the size and complexity of our project. A 'locked down' master version of the consolidated NVIVO interview data base is now held at The George Institute, Sydney. Team members undertaking further analysis will work from copies of this master version.

Two IMPAKT interviewers completed all coding; each read every interview at least once. As with the actual interviewing, the IMPAKT team was of the view that immersion in the task of coding was integral to the quality of interpretations of the consolidated interview material. Coding and entering associated data for 383 interviews took approximately 6–8 person/months and was completed in February 2007. During this process the two coders spoke weekly by phone, comparing progress, but more importantly noting issues of misalignment which could potentially complicate the necessary final merge of both sets of coded interviews.

The collection of education materials remains to be analysed.

#### Outputs from NVIVO

Demographic and other descriptive information was included in the NVIVO project. While the inclusion of this participant information is invaluable for the sorting and targeted searching of the interview material, due to NVIVO's limited statistical functionality, this demographic and descriptive data was exported for analysis into SPSS 15.0 for Windows (Chicago, Illinois).

### Other kinds of data

As noted above, the IMPAKT team undertook several other activities at each service centre besides interviewing. Each was intended to better understand the local system, the situation of patients and the nature of the programs provided.

#### Mapping a patient's journey

The system of delivering renal services – while similar in essentials – is complex and varies from location to location. A critical feature of the local system is the way it articulates with other service centres involved in kidney patients' care, including on the one hand the transplant unit and, on the other, the relation to the residential location and/or treatment site of the patient. Transplants are carried out in metropolitan hospitals in southern, urban Australia. Patients, on the other hand, are receiving dialysis services in locations everywhere including metropolitan, urban, regional, remote and very remote Australia (Table 1, Figure 2). The nature of the local health services delivery system is a crucial contextual dimension for the commentary of both patients and staff. IMPAKT required an overview of each local service system.

We approached this task using an adapted version of 'process mapping' described by the Modernisation Agency of the National Health Service (NHS) of the United Kingdom [52]. The objective of the exercise was to lay out a schematic map of a patient's journey through the local system starting from an agreed entry point and moving through to transplant. The journey was mapped from the orientation of a patient, i.e. what happens to a patient. It therefore differs significantly from the more familiar 'clinical care path' although there would be overlap. To illustrate with one example: when the *patient *journey is mapped, it emerges clearly when patients may be required to travel hundreds of miles back and forth between services. It then becomes clear where there are bottlenecks and delays for patients. Since the research focus was on the experience of *Indigenous *patients, the majority of the maps tracked an Indigenous person and mostly one from a regional or remote location. Along with charting a patient's movements/activities, step by step, the categories of staff involved at each step were jotted on the map, as were issues of concern identified by staff. Given the level of system complexity, it was impossible to map in any detailed sense the myriad activities of ESKD management – nor is this the purpose of 'process mapping'. Nevertheless, the maps constructed in these sessions provided an additional layer of information on the local situation.

IMPAKT adapted process mapping into a more 'ethnographic' field technique than is usually the case. In qualitative research terms the process mapping activity was essentially a type of 'focus group'. It was usually undertaken with staff in the first days after arrival at a service centre, often over a light lunch provided by the project. Judged on the level of attention, it was an engaging activity and staff were soon plotting the journey, arguing the point and discussing sequences, issues and systems. For the IMPAKT interviewers as 'outsiders', the session was a valuable window onto staff relations at the site, as well as a quick introduction to local staff and their tasks as they related to our primary research interests [see Additional file 23].

The map itself is a snapshot of staff perceptions of how things were organised locally and how the local service centre connects to other service centres, particularly the transplant units. The mapping sessions were documented in the maps themselves and in brief field notes. On several occasions staff later commented on how the exercise had clarified for them aspects of their own system and processes, how it had brought the segments of day-to-day operations into a whole. Two service centres requested copies for their own internal planning and management use.

Following the NHS method, the first version of each map was constructed on one or more large sheets of paper, using various coloured 'post-it labels' and a variety of coloured pens [see Additional file 24]. The completed map – typically a metre or so in length – was then re-drawn by the IMPAKT interviewers and returned within a day or so to the workplace where it was attached to a wall for staff review and further comment [see Additional file 25]. Process maps were completed at 13 sites in five states, including 4 metropolitan hospitals with transplant units, 5 regional hospitals and 3 satellite dialysis units. In all cases, it was an interactive group activity lead by staff primarily from the local reference group.

As a point of comparison, two Indigenous patients were also invited to describe their own 'patient journey' using a similar mapping process. The process was not comparable insofar as the patients worked individually and privately on their maps. However, approached as a focus group activity involving the patient and relevant family, the process mapping technique has potential to reveal (and clarify) Indigenous perceptions of process, emphasis and priorities. Later publications will describe the process map data set more fully.

#### Informing and educating patients

Patient education in general does not have a sharp disciplinary focus in Australia and literature describing best practice relates primarily to the situation in the United States. Even so, there is a paucity of research concerned with education for minority and/or other culturally and linguistically diverse (CALD) groups [53-55]. Education for kidney patients in particular is similarly poorly defined. So, for example, in relation to 'pre-dialysis' education (often delivered *after *people have actually started dialysis i.e. post-dialysis), the relevant Australian national guideline (CARI) provides no recommendations or guidance for patient education on the grounds that there is no available evidence of the required standard [56]. The CARI (Caring for Australians with Renal Impairment) Guidelines, an evidence-based program that commenced in 1999, is supported by the Australian and New Zealand Society of Nephrology (ANZSN) and Kidney Health Australia (KHA).

The Interview study targeted nursing staff with primary responsibility for educating patients starting dialysis or patients considering transplant. Prior to patients beginning dialysis, they undergo (theoretically at least) a program informing and educating them on their illness and treatment options. This is usually the most comprehensive information session(s) for the majority of patients and is therefore a fundamental aspect of patients being well informed. IMPAKT interviewers sought out these particular educators, often termed Chronic Kidney Disease (CKD) Educators, at all service centres. The interview schedule for CKD educators included the (common) component on Information and Communication but also involved a questionnaire concerning the delivery of patient education in that setting [see Additional file 26]. Topics included:

• methods of assessing patients' learning preferences and capacity;

• the program structure including design responsibilities;

• program processes including personnel and documentation;

• interpreter use; and

• program setting – locations and support resources.

The Education questionnaire was designed to be completed by educators. But it was soon clear that CKD educators found it somewhat intimidating with its assumptions of a programmatic (as opposed to an individualistic) approach to educating patients. Again, a conversational approach whereby questions were answered as part of a discussion diminished the negative aspect of the interaction. These conversations were taped and transcribed.

Transplant education processes are organised differently to CKD education. The large metropolitan transplant units run regular, formal day/half day patient education forums in the metropolitan area only. Attendance at these sessions is often a compulsory element of the local pre-transplant preparation, or is the only interactive education provided directly to patients awaiting transplant. However, it was not easily accessed by patients from beyond the metropolitan area and was totally inaccessible for those in remote locations. Non-English speakers were similarly disadvantaged. Some regional hospitals had a 'transplant coordinator' position which involved overseeing the workup processes preparing patients for transplant, post-transplant follow up and some education responsibilities. Several transplant coordinators were interviewed but did not complete the education questionnaire as it was less relevant to their work situation.

Another distinct stream of 'patient education' within larger service centres is organised around teaching patients home therapies – either home haemodialysis or peritoneal dialysis. As noted, the allocation of these roles against actual positions varied widely from place to place, although trainers for home therapies tended to be full time positions.

A final component of the investigation of education processes at each site included documenting the range and type of resources provided to support patient education. Educators were invited to show and discuss the resources they use, including videos, pamphlets, worksheets and so on. As well as collecting many dozens of these, including information packages assembled for patients, a checklist was compiled at each site [see Additional file 27] and photographs were taken. Materials actually used by educators were noted separately from those available freely as 'self serve' in various locations around the service centres. A selection of the items collected will be assessed using a method to evaluate materials for use with low literacy patients [54].

Complementary information was collected from patients including years of schooling, self-reported English reading capability, ownership of a computer and use of the internet for self education about kidney disease. Patients were also asked to comment on whether they felt they had been sufficiently informed about their illness, about treatment options and whether they believed they could access information.

#### Pre-transplant patients' knowledge

Adapting from earlier research reports [33,57], IMPAKT designed an exercise to assess Indigenous pre-transplant patients' knowledge about transplant. The activity involved patients who believed themselves to be on the transplant list, sorting a series of 12 short statements according to whether they were 'true' or 'not true'. The team nephrologists, who had worked with Indigenous patients from remote communities, reviewed the content of the statements and the interviewing team and the nephrologists collectively worked on the final plain English versions of the statements. It emphasised less the clinical and medical dimension of transplant and more the psycho-social, risk and general understanding aspects. The set of statements included items with broader implications for understanding patients' perspectives, for example the statement: "*My kidneys will get better"*.

Each of the 12 statements was produced as a laminated strip [see Additional file 28]. The bundle of strips was given to the patient and they were invited to give the interviewer those strips they thought to be 'not true'. Where literacy was an issue, the interviewer read each card as it was placed down for the patient assessment. The score was recorded on the reverse of the Record of Interview Sheet.

The card sort was ultimately less useful than was hoped with Indigenous patients. For those with no previous familiarity with such activities as well as limited English, the exercise proved confusing. Worse, it was potentially a source of misinformation as some patients began pondering statements that were untrue. On the other hand, for Indigenous people for whom English and/or literacy was not an issue, the content was relatively undemanding and they sorted the cards quickly and correctly. The exercise highlighted how issues of language, literacy and lack of a shared understanding of *the purpose *of the interaction, in combination, might distort and mask the understandings of participants. The research methods themselves become confounders.

#### Field observations: diaries and photographs

The team anthropologist (JD) maintained a diary both prior to and during the field work. The diary included the author's observations on different field sites and records unsolicited comments, including suggestions and criticisms made by various individuals as well as notes on service centre layouts. Comments and experiences reported by other team members were also recorded. It was also a tool for efficient local work – recording names, job titles, contacts, participants in meetings held, notes on meetings and so on. In the work place, a paper-based diary was less obtrusive and more convenient than a laptop. There are five field diaries comprising around 600 numbered pages of hand written notes, as well as an additional electronic diary set up within NVIVO. The diaries – both hand written and electronic – are linked into the NVIVO database.

Photographs were taken at every opportunity, particularly in relation to location and service centre settings, renal equipment, educational resources and the like. We rarely took photographs of individual patients or staff, and only with permission of the individuals concerned. On the whole, Indigenous ESKD patients, particularly those from regional and remote areas, preferred not to profile their individual health concerns in public forums. High rates of death associated with ESKD and cultural protocols constraining the display of names, images and sounds of deceased persons make photography and/or film a sensitive matter requiring a specific consent process. However, in our newsletter, we did feature one or two Aboriginal patients willing to share their experiences of various treatments.

### Resources: timeline and funding

The IMPAKT research program got underway in early 2004 and is now in its concluding phase. Costs were somewhat increased through unpredicted delays as well as expanded data collection. Significant delays were associated with:

• the multiple, complex ethics approval processes; and

• acquiring institutional licence access (for 2 different locations) to the newly-released NVIVO 7.

The inclusion of an expanded number of service centres (up from a planned 8 hospitals to 26 hospitals and dialysis service centres) increased the number of staff interviews from a planned 30 to 114. On the other hand, recruiting Indigenous patient participants was enhanced by including the satellite dialysis centres as well as the large hospitals; it would otherwise have been difficult to meet our target for Indigenous patients.

The project was funded by Australia's National Health and Medical Research Council (project grant #236204). Additional funding was obtained through a consultancy related to renal health services for the Australian Health Ministers' Advisory Council [58]. One team member was partially funded through an Australian Postgraduate Research Scholarship. The IMPAKT project was an approved 'in-kind' project of the Co-operative Research Centre for Aboriginal Health (CRCAH). The CRCAH contributed directly to the project feedback processes, to the NVIVO training for the Indigenous Researcher and, indirectly, to the support of the team anthropologist (JD). NVIVO training for JD was funded by Menzies School of Health Research.

## Discussion

The IMPAKT Interview study has a national scope, an inter-disciplinary approach and used qualitative methods to investigate an example of significant health inequality affecting Indigenous Australians. It has a large, diverse group of Indigenous participants who give evaluative comment on their health services. The study includes commentary from a group of specialists, nursing and other staff and the research has been conducted in a diverse range of treatment centre/workplaces. The IMPAKT Interview study protocol may contribute to improvements in flexible design research practice with Indigenous and other hard to reach or vulnerable populations, both in Australia and elsewhere.

Several factors contributed to the strength of the Interview study design. As well as being personally important for participating individuals, the central research question was considered significant by key stakeholder groups. The multi-disciplinary research team included experienced 'insiders' in relation to key stakeholder groups and allocated substantial time, thought and energy to maintaining a collaborative ethos between the research team and the many participant groups. The study design featured triangulation – conventionally understood to increase the validity of qualitative research – at several different levels, including sources of data (structured and semi-structured interviews, focus groups and direct observations systematically recorded in diaries) and categories of commentators (staff, Indigenous and non-Indigenous patients, specialists). In the context of the broader IMPAKT research program, which has investigated the same question using quantitative methods, there is a further level of triangulation.

Despite hearing from a diverse range of Indigenous patients in different locations, with different social histories, and using different treatments, it is likely that the Indigenous cohort in the IMPAKT study represents the more confident segment of the total Indigenous ESKD population. Patients who are non-English speaking and/or who are shy, lacking in confidence, angry or confused are less likely to have voluntarily engaged in this research process. The IMPAKT interview study data is therefore likely to *understate *the level of difficulty some ESKD patients experience.

Although most reference groups included Indigenous staff there was a relatively low level of involvement of other local Indigenous health and advocacy agencies. With some notable exceptions, renal service centres themselves did not have established relationships with such Indigenous agencies. IMPAKT will therefore require a separate and specifically designed strategy to effectively disseminate the results of the IMPAKT Interview study to Indigenous patients, families and their advocates.

The study is limited insofar as the commentaries remain a partial account of the interviewee experience and of the service system processes; they are *reported *perceptions, attitudes, behaviours and experiences. Multiple points of view reduce the potential for bias, but there remains a gap between *accounts *of behaviour and processes, and *observations *of the actual behaviour and processes themselves. Nevertheless, the viewpoints of key participants are primary influences on patient outcomes and system processes and can usefully inform potential interventions.

Overall success hinges first, on the quality and relevance of IMPAKT's research outputs and second, on fulfilling reciprocal obligations to provide feedback to participating service centre patients and staff. Both of these are not yet completed. However, there has been considerable concurrent activity in relation to both policy and practice, not least of which was completing a major national consultancy for the Australian Health Ministers' Advisory Council concerning strategies to improve access to renal replacement therapies for Indigenous people in regional and remote Australia [58]. There has been intense activity since 2004 by all team members at relevant professional conferences including those for renal nurses, nephrologists and transplant surgeons. Perhaps more importantly, the core team continues to develop networks with key stakeholder groups to establish the pre-conditions for possible systemic change.

## Competing interests

The author(s) declare that they have no competing interests.

## Authors' contributions

JD participated in the design of the IMPAKT Interview study protocol, coordinated and participated in data collection, management and analysis and drafted the manuscript. AC conceived of the study, participated in its design, conducted nephrologists' interviews and participated in data analysis. JC participated in the overall management and design of the IMPAKT program, provided statistical and other technical assistance during data collection and participated in data analysis. CP participated in study design, coordinated the Indigenous community engagement component, participated in data collection, management and analysis. KA coordinated data management and participated in data collection as well as data analysis. PS participated in design, provided specific clinical expertise and participated in data analysis. All authors participated in the drafting and/or critical revision of the manuscript and approved the final version to be published.

## Pre-publication history

The pre-publication history for this paper can be accessed here:



## Supplementary Material

Additional file 1 PDF, IMPAKT Reference group meeting proforma; Notes for each reference group meeting preceding interviewing at each site. Click here for file

Additional file 2 PDF, Poster "Are You On Dialysis" x 2; A3 size posters sent ahead to treatment centres; each uses a recognizable local icon. Click here for file

Additional file 3 PDF, IMPAKT Newsletter; Example of the newsletter produced during the project. Click here for file

Additional file 4 PDF, IMPAKT Project Information Sheet – professionals; Project explanation provided to and discussed with staff and other professionals. Click here for file

Additional file 5 PDF, IMPAKT Project Information Sheet – patients; Project explanation provided to and discussed with patients, carers, families. Click here for file

Additional file 6 PDF, IMPAKT Consent Form; Proforma for those agreeing to participate includes various conditions. Click here for file

Additional file 7 PDF, IMPAKT Renal nursing staff interview (IMP Q3); Questions put to renal nurses in dialysis units. Click here for file

Additional file 8 PFD, IMPAKT: Social support staff interview (IMP Q6); Questions put to staff involved in social support for kidney patients. Click here for file

Additional file 9 PDF, IMPAKT Nephrologist interview (IMP Q5); Questions put to nephrologists. Click here for file

Additional file 10 PDF, IMPAKT Patient Interview – full version (IMP Q4); Questions put to patients (as appropriate). Click here for file

Additional file 11 PDF, IMPAKT Patient Interview -outline – plain English (for patients); A version of the patient questions in more accessible language. Click here for file

Additional file 12 PDF, IMPAKT Patient Interview – prompt points only; A version of the patient questions showing a list of prompts for interviewer. Click here for file

Additional file 13 PDF, Peritoneal Dialysis cycler machine; Photo of peritoneal cycler machine in a home therapies instruction setting. Click here for file

Additional file 14 PDF, A regional hospital based dialysis unit; Photo. Click here for file

Additional file 15 PDF, Remote area Aboriginal Community Controlled Dialysis Unit; Photo of plaque explaining purpose of facility. Click here for file

Additional file 16 PDF, Remote area Indigenous home haemodialysis facility; Photo. Click here for file

Additional file 17 PDF, Dialysis chair and haemodialysis machine; Photograph. Click here for file

Additional file 18 PDF, Dialysis needles; Photo – needles that are inserted into fistula at every dialysis session. Click here for file

Additional file 19 PDF, Small dialysis unit following treatment sessions; Photo. Click here for file

Additional file 20 PDF, IMPAKT Record of Interview – Patient; Details of interview sociodemographic data of interviewee. Click here for file

Additional file 21 PDF, IMPAKT Record of Interview – Renal Staff; Details of interview; socio-demographics of interviewee; ranking of hospital services. Click here for file

Additional file 22 PDF, IMPAKT Record of Interview – Patient Educators; Details of interview, socio-demographics of interviewee; notes on education programs. Click here for file

Additional file 23 PDF, Renal staff working on a 'patient journey' process map; Photo. Click here for file

Additional file 24 PDF, Completed Original version of 'patient journey' process map; Photo. Click here for file

Additional file 25 PDF, Re-drafted version of 'patient journey' process map; Photo. Click here for file

Additional file 26 PDF, IMPAKT Questionnaire: Educational (Patient) Programs Description IMP Q1; Questions put to CKD and other patient educators. Click here for file

Additional file 27 PDF, Patient Educational Resources Checklist; A sheet used to record resources available for use in patient education including 'self serve' information. Click here for file

Additional file 28 PDF, IMPAKT card sort transplant knowledge; Content of cards; these were transferred to coloured card, cut into strips and laminated. Click here for file
