# Supplementary material for: Study Protocol – Improving Access to Kidney Transplants (IMPAKT): A detailed account of a qualitative study investigating barriers to transplant for Australian Indigenous people with end-stage kidney disease
Source: BMC Health Serv Res. 2008 Feb 4;8:31. doi: 10.1186/1472-6963-8-31 (PMC2275237; doi:10.1186/1472-6963-8-31)
Supplement: Additional file 1 — PDF, IMPAKT Reference group meeting proforma; Notes for each reference group meeting preceding interviewing at each site. [file 1472-6963-8-31-S1.pdf]

REFERENCE GROUP<br>SITE:<br>DATE:	impakt – Improving Access to Kidney Transplants

The purpose of the IMPAKT Reference Group is to provide:

1. a point of regular contact between the research team and key stakeholders at each participating site;
2. a forum to raise and discuss issues relevant to the conduct of the research in the local area
3. an avenue for short-term feedback on IMPAKT progress and outcomes;

Reference Group Membership

Representatives of the key stakeholder groups may include renal staff from participating sites, patients, and local Aboriginal Health Service representatives. The membership size is flexible, with an emphasis on maintaining an effective working size (i.e. not too big). We particularly encourage the involvement of site-based Aboriginal/Torres Strait Islander staff involved with renal patient care.

Processes

Meetings will be few and brief. They will be:

- irregular, face-to-face meetings, convened by IMPAKT or local members as required;
- informal but with a clear agenda
- documented by IMPAKT team members.

Issues for first Reference Group meeting

Before the IMPAKT interviews start the reference group will meet to:

- agree on suitable process for (volunteer) staff interviews;
- moving around the site/s - specific instructions/protocols
- agree on suitable processes for patient contacts –
    - availability of local broker/consultant?
    - need for interpreters; local languages

- counselling arrangements (as per ethics applications)
- agreement on protocols for managing issues of concern or complaints that may arise within the interview process at the sites.
- feedback arrangements (when, how)
