# Supplementary material for: Study Protocol – Improving Access to Kidney Transplants (IMPAKT): A detailed account of a qualitative study investigating barriers to transplant for Australian Indigenous people with end-stage kidney disease
Source: BMC Health Serv Res. 2008 Feb 4;8:31. doi: 10.1186/1472-6963-8-31 (PMC2275237; doi:10.1186/1472-6963-8-31)
Supplement: Additional file 2 — PDF, Poster "Are You On Dialysis" x 2; A3 size posters sent ahead to treatment centres; each uses a recognizable local icon. [file 1472-6963-8-31-S2.pdf]

impakt
Improving Access to Kidney Transplants

ARE YOU ON DIALYSIS?

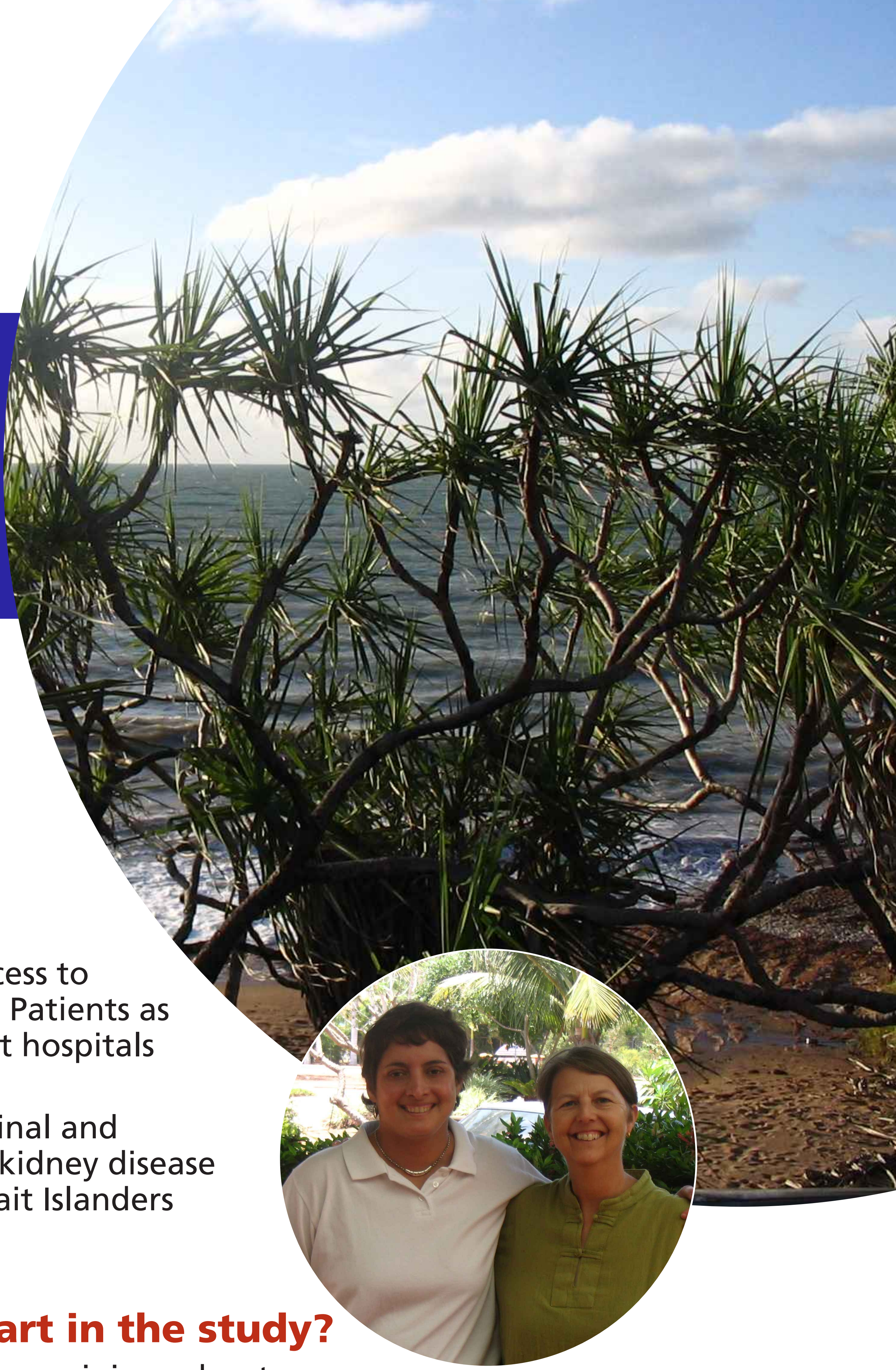

We invite you to tell us about your experiences

What is IMPAKT about?

IMPAKT is a study about improving access to kidney transplants for suitable people. Patients as well as staff will be joining the study at hospitals around Australia.

We are very keen to hear from Aboriginal and Torres Strait Islander patients because kidney disease affects more Aboriginal and Torres Strait Islanders than other people.

What will it mean if I take part in the study?

Interviewers from our team will ask your opinions about treatments, about the kind of information you get and any difficulties you have. We would like to record your experiences.

This study does not involve any medical treatments. There is no payment for this.

What is the point of taking part in this study?

You will help us understand the barriers to transplant so they might be moved. All suitable people will then have a better chance for a transplant.

When will we be here?

Our interviewers, Jeannie Devitt and Cilla Preece, will be in the Nightcliff Renal Unit & Royal Darwin Hospital between

9 - 20 May 2005

THE GEORGE INSTITUTE
for International Health

If you are interested to participate in this study, please ask a nurse in this renal unit for an information sheet or contact: Kate Anderson

Ph: (02) 9993 4574 or kanderson@thegeorgeinstitute.org

impakt
Improving Access
to Kidney Transplants

ARE YOU ON DIALYSIS?

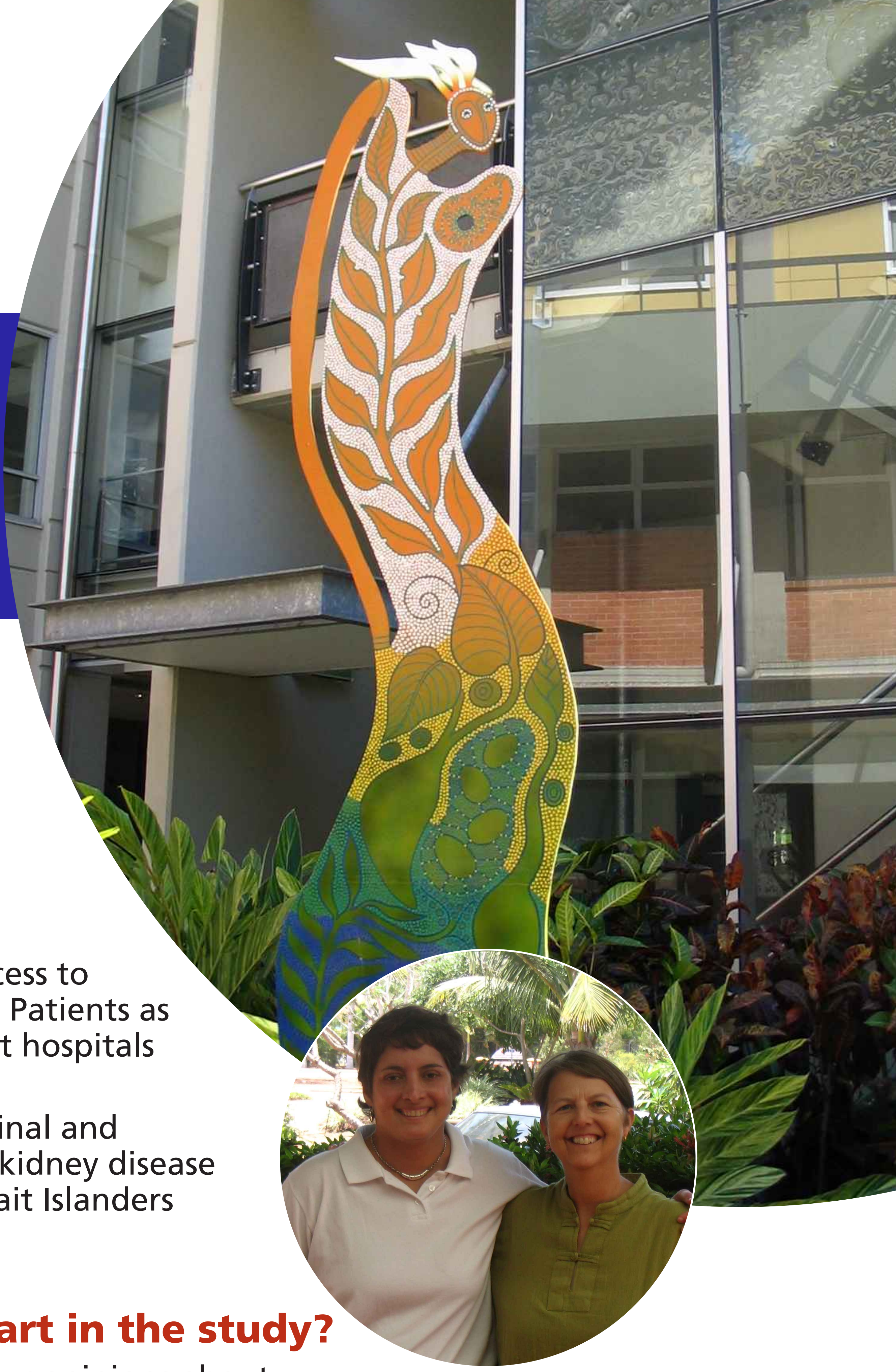

We invite you to tell us about your experiences

What is IMPAKT about?

IMPAKT is a study about improving access to kidney transplants for suitable people. Patients as well as staff will be joining the study at hospitals around Australia.

We are very keen to hear from Aboriginal and Torres Strait Islander patients because kidney disease affects more Aboriginal and Torres Strait Islanders than other people.

What will it mean if I take part in the study?

Interviewers from our team will ask your opinions about treatments, about the kind of information you get and any difficulties you have. We would like to record your experiences.

This study does not involve any medical treatments. There is no payment for this.

What is the point of taking part in this study?

You will help us understand the barriers to transplant so they might be moved. All suitable people will then have a better chance for a transplant.

When will we be here?

Our interviewers, Jeannie Devitt and Cilla Preece, will be in Cairns Base Hospital & Calvary Hospital Renal Units between

22 February - 11 March 2005

THE GEORGE INSTITUTE
for International Health

If you are interested to participate in this study, please ask a nurse in this renal unit for an information sheet or contact: Kate Anderson

Ph: (02) 9993 4574 or kanderson@thegeorgeinstitute.org
