# Supplementary material for: Study Protocol – Improving Access to Kidney Transplants (IMPAKT): A detailed account of a qualitative study investigating barriers to transplant for Australian Indigenous people with end-stage kidney disease
Source: BMC Health Serv Res. 2008 Feb 4;8:31. doi: 10.1186/1472-6963-8-31 (PMC2275237; doi:10.1186/1472-6963-8-31)
Supplement: Additional file 3 — PDF, IMPAKT Newsletter; Example of the newsletter produced during the project. [file 1472-6963-8-31-S3.pdf]

IMPROVING ACCESS TO KIDNEY TRANSPLANTS

IMPAKT newsletter

impakt
Improving Access
to Kidney Transplants

Issue 5 | November 2006

A (kidney) health expo in Alice Springs

In early June Dr Alan Cass, IMPAKT's Chief Investigator & Director of the Renal Division at The George Institute, was keynote speaker at the Health Expo held in Alice Springs. The event was sponsored by Transplant Australia and AMGEN. Several local organizations, particularly Central Australian Aboriginal Congress were involved in the Expo, held on a beautiful sunny Centralian winter day at Blatherskite Park.

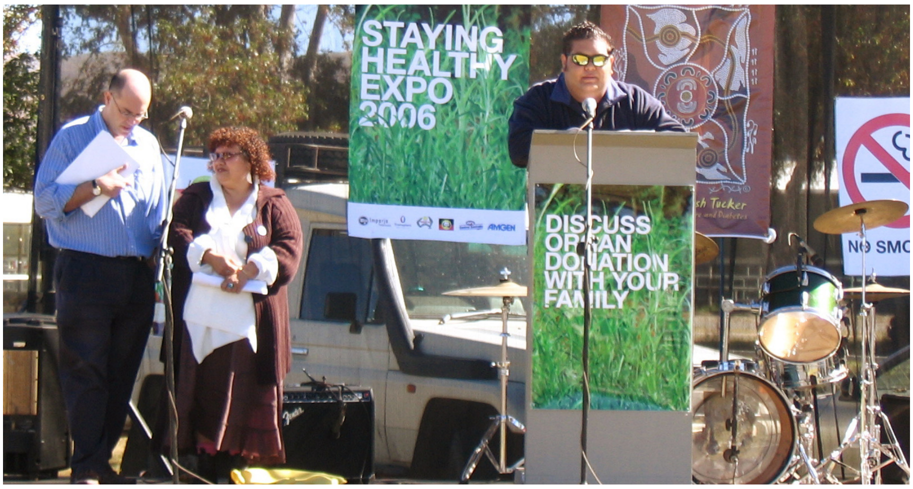

Stephanie Bell and Alan Cass prepare to open the Health Expo in Alice

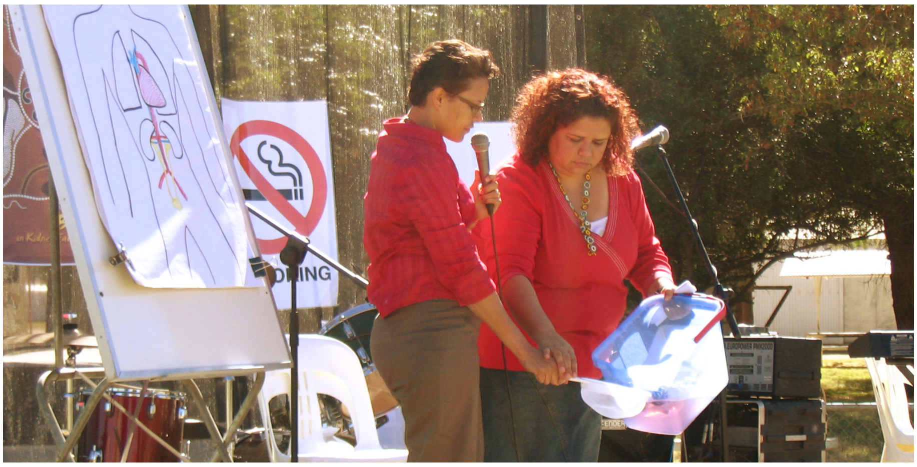

Jacqui Hughes and Tina Whap demonstrating how kidneys work as filters

New National Service Guidelines for Renal Services to Remote Australia

In 2003, as part of the process of renewing the Australian Health Care Agreements, the National Health Reform Agenda was developed. One priority area identified was addressing the situation of patients in remote areas – primarily Indigenous people – requiring renal care.

Following a comprehensive review of renal services in remote areas in 2004, the Australian Health Ministers Advisory Council endorsed a set of principles and identified national renal service guidelines for remote Australia as a priority strategy. Ms Gillian Gorham, of the NT Dept of Health and Community Services, coordinated a cross-jurisdictional working party with relevant expertise to develop draft guidelines. These guidelines, which were widely distributed within each jurisdiction for comment, were subsequently endorsed at a well-attended meeting of stakeholders including government, non-government, Aboriginal community-controlled and consumer advocacy groups. The document — The National Service Guidelines for the Management of Dialysis and Kidney Transplantation in Remote Australia — is now an important reference document for clinicians and other renal service providers. It outlines best practice approaches and includes performance measures. This important document supports improved clinical outcomes for people in remote Australia particularly Indigenous people.

(This document should (shortly) be available on your state/territory department website and on the Kidney Health Australia website.)

THE GEORGE INSTITUTE
for International Health

Cooperative Research Centre for
Aboriginal Health

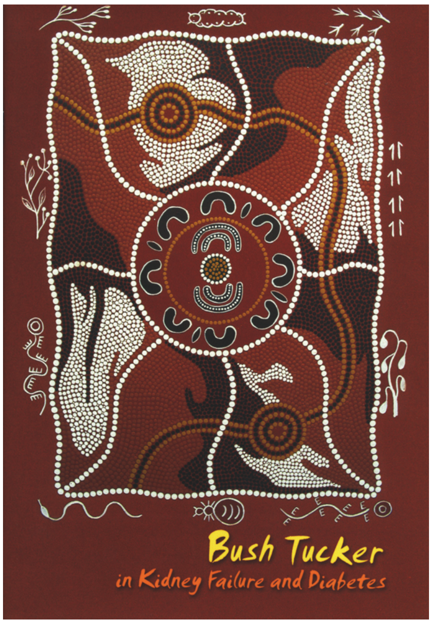

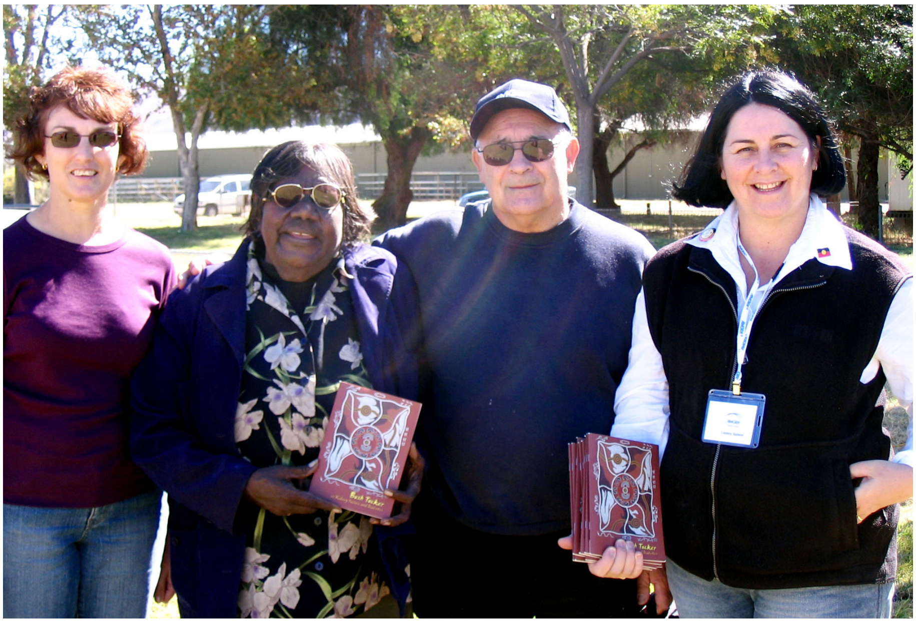

Above: Lesley's father, Herbert Elvin, did the art work for the cover of the Bush Tucker booklet

Above right: R to L: Ms Lesley Salem, Herbert Elvin, MM Turner and Elizabeth Thompson

Right: Jimmy Little and co star entertaining visitors at the Health Expo.

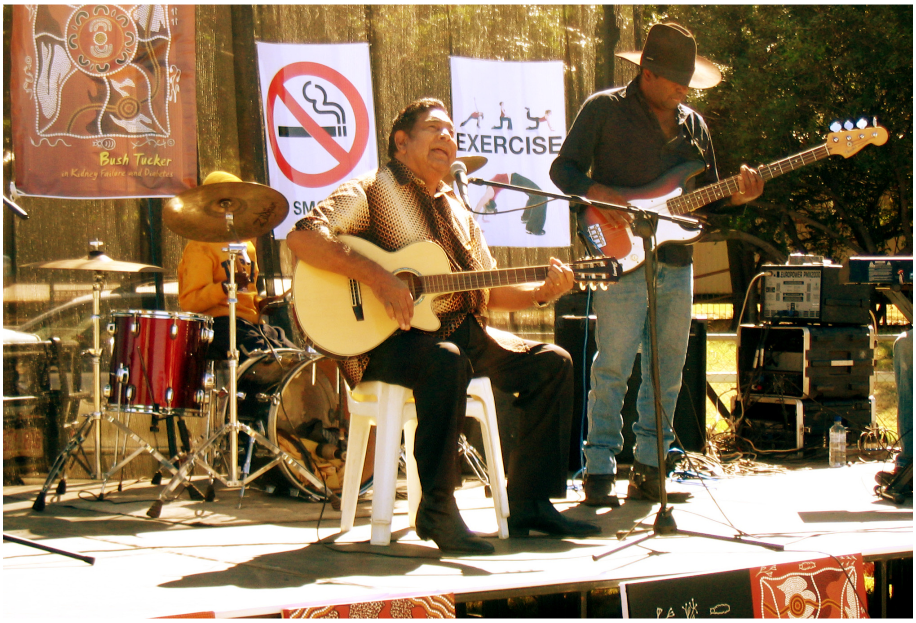

Bush Tucker for kidney patients

One of the main events of the Expo was the launch of Ms Lesley Salem's booklet on bush foods for kidney patients. Lesley is an Indigenous Nephrology Nurse Practitioner with over twenty years experience in renal nursing. She currently works for the Lower Hunter New England Health Service. Ms Margaret Mary Turner — a senior Eastern Arrernte woman and local bush tucker champion — launched her book Bush Tucker in Kidney Failure and Diabetes. Lesley is a co-author of a second book also launched at the Expo — The health management plan for end stage kidney disease. The book supports achieving the best possible End Stage Kidney Disease care.

Getting to know more about kidneys

The crowd was introduced to the kidneys and given a lively demonstration on how they work – or don't work – by Dr Jaqui Hughes and her sister Ms Tina Whap, a talented team of young Indigenous women. Jaqui is currently a trainee nephrologist at the Prince of Wales Hospital and Tina is the Clinic Nurse at Nyirrpi, a remote community north west of Alice Springs. We hope to see more of this 'direct action learning' in areas where kidney disease is such a problem for Indigenous communities.

Presentations at the combined ANZSN-RSA Conference 2006

We presented an early analysis of some of the interview data at the recent Renal Society of Australasia Annual Conference in Melbourne. We looked at nurses' responses to the question: How – if at all – does ethnicity or cultural difference influence the way you manage patients? The kinds of differences most commonly thought to influence management were:

- Language, literacy & communication issues; and
- Perceived level of social need.

These will be important themes in the continuing analysis. The interviews also suggest that in areas of great disadvantage, where many patients have high levels of need — including such things as financial, housing, transport, social support etc — responding to 'social needs' can be a source of work stress.

Three posters were presented at the Australia and New Zealand Society of Nephrology Annual Scientific Meeting in Melbourne. The posters explored the findings from the nephrologists' survey concerning decision-making about suitability for transplantation and how renal unit guidelines handle issues such as compliance and smoking status. There is significant variation across Australia in unit guidelines and also in the views of providers and patients regarding how the system works, or fails to work, to facilitate appropriate patients receiving transplants. We will feed this information back to each of the local sites participating in the study.

A new appointment

Earlier this year, Cilla Preece, one of our key IMPAKT team members and lead advisor for our Indigenous community participation processes, was elected to the WuChopperen Health Service Board. WuChopperen provides health services to the Aboriginal and Torres Strait Islander communities of the Cairns area. Congratulations Cilla!

Stop Press - WuChopperen Health Service have just been named as 2006 winners of the Indigenous Governance Awards. Congratulations WuChopperen!

Attending Communication, Medicine & Ethics (COMET) Conference 2006

One of our IMPAKT team members, Ms Kate Anderson, presented at the Communication, Medicine & Ethics (COMET) Conference in Cardiff, UK in June. Kate presented on the topic of patient compliance – focusing on how being labeled 'non-compliant' can prevent patients from accessing kidney transplants and yet how difficult a concept patient compliance is to define and measure.

This presentation initiated some lively discussion about the use of compliance-labelling in clinical settings. We look forward to Kate presenting some more findings in Australia relating to this problematic issue.

If you would like a copy of this presentation, please email kanderson@george.org.au.

Canadians Improving Aboriginal Access to Kidney Transplantation

Aboriginal Australians and Aboriginal Canadians share very high rates of end-stage kidney disease. Unfortunately, they also share relatively low rates of kidney transplantation. It is likely that similar barriers are experienced by both of these groups, which may contribute to these low rates of transplantation.

A Canadian kidney specialist and public health researcher, Dr Karen Yeates from Queens University, is conducting a similar study to IMPAKT within the Canadian context. This Canadian study is called Canadians Improving Aboriginal Access to Kidney Transplantation (CAN-IMPAAKT).

CAN-IMPAAKT comprises several sub-studies, including a series of in-depth interviews with renal staff from across Canada. One of our IMPAKT team members, Ms Kate Anderson, is currently spending six months working in Canada with Dr Yeates.

It is hoped that this type of comparative health research between Canada and Australia will help to produce innovative approaches to improving the health of the Indigenous people in both countries.

Jimmy Little Foundation launches

After two years of dialysis and a kidney transplant in February 2004, Jimmy Little realised the importance of all Australians being able to access quality health care no matter where they live and wanted to make a concrete contribution to improving indigenous health, particularly kidney health, across regional and remote Australia.

In mid 2006, Jimmy joined forces with a group of concerned business people to create the Jimmy Little Foundation (JLF); which aims to work for a healthier future for Indigenous Australians. One key area the JLF is supporting is "Return to Country" programs so those who have to move far from home for treatment can return regularly to connect with family, community, Country and culture.

Jimmy continues to travel Australia attending community events, health seminars and music festivals. He is the patron of The Indigenous Doctors Association of Australia, an ambassador for The Fred Hollows Foundation and Kidney Health Australia. He also is an ambassador for literacy and numeracy with the Federal Department of Education Science and Training, reinforcing positive messages to schoolchildren across Australia.

The JLF will evaluate submissions from communities in need and help the communities achieve the goals they have set for themselves. The Foundation wants to help make it easier for communities to take control of their own projects and build their capacity to improve their situation.

For more information please visit the Jimmy Little Foundation website at www.jlf.org.au.

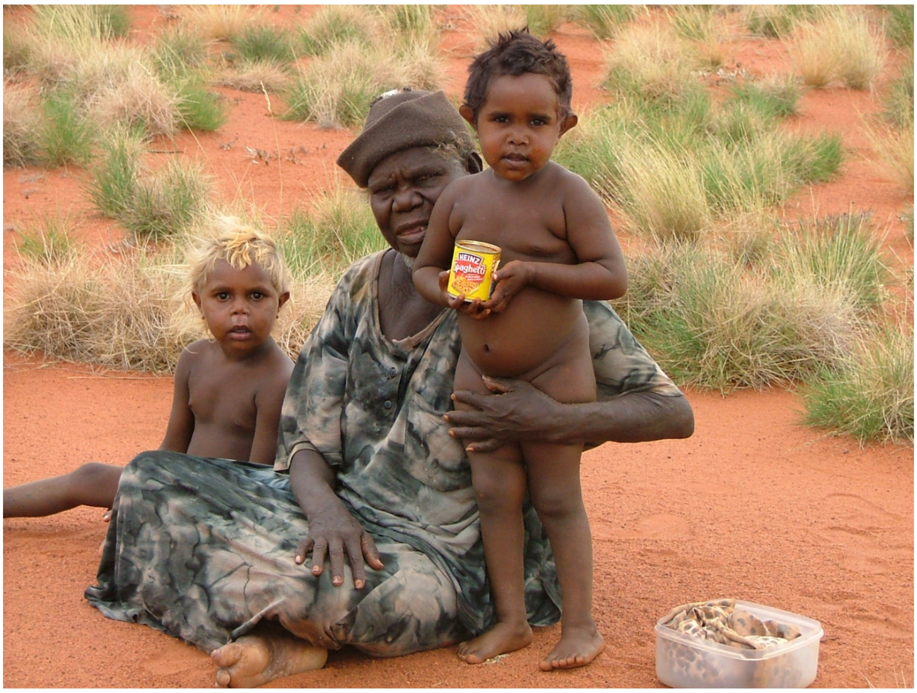

Mary Tolson, with her grandchildren, in Kintore during her 4th trip home made possible through community-based dialysis established through WDNWPT

Western Desert News

This month the WDNWPT celebrates two years of getting Anangu home to Kintore on dialysis. In June this year, Sothebys and Papunya Tula artists auctioned a major painting in Paris and held an exhibition in London to assist the organisation to raise further funds to continue their work. Meanwhile the WDNWPT Committee is working with the Commonwealth Government on a 'Shared Responsibility Agreement' to expand their services and provide resources for a greater emphasis on early intervention and prevention of kidney disease in the Western Desert.

To find out more please ring Sarah Brown on (08) 89530002, or contact her by email at wdnwpt@bigpond.net.au

Send us any items of interest, snippets and tips

By post: IMPAKT
The George Institute
PO Box M201 Missenden Road, Camperdown, Sydney NSW 2050

By email: impakt@thegeorgeinstitute.org
