# Supplementary material for: Study Protocol – Improving Access to Kidney Transplants (IMPAKT): A detailed account of a qualitative study investigating barriers to transplant for Australian Indigenous people with end-stage kidney disease
Source: BMC Health Serv Res. 2008 Feb 4;8:31. doi: 10.1186/1472-6963-8-31 (PMC2275237; doi:10.1186/1472-6963-8-31)
Supplement: Additional file 4 — PDF, IMPAKT Project Information Sheet – professionals; Project explanation provided to and discussed with staff and other professionals. [file 1472-6963-8-31-S4.pdf]

We invite you to participate in the IMPAKT study

The George Institute
ABN 90 085 953 331

Level 10 King George V Building
Royal Prince Alfred Hospital
Missenden Road Camperdown
Sydney NSW 2050 Australia

PO Box M201 Missenden Road
Sydney NSW 2050 Australia

Telephone +61 2 9993 4500
Facsimile +61 2 9993 4501

impakt@thegeorgeinstitute.org
www.thegeorgeinstitute.org

Purpose of the IMPAKT study

People with end stage renal disease (ESRD) need dialysis or a transplanted kidney to stay alive. Transplantation is the best treatment for many people with ESRD. Transplantation is a sensitive issue and we understand that some people may prefer not to have this treatment. At present, Aboriginal and Torres Strait Islander Australians develop ESRD at up to nine times the rate for other Australians. But, although they are over-represented among the ESRD population, Aboriginal and Torres Strait Islanders have approximately one third the chance of receiving a transplant as other Australians.

The IMPAKT study aims to improve access to kidney transplants for Indigenous Australians by better understanding the 'steps' and 'gates' involved on the pathway to receive a kidney transplant

IMPAKT study description

In order to receive a kidney transplant, a dialysis patient must negotiate the following steps:

Dialysis Patient →	Step 1<br>Deemed medically or otherwise suitable	Step 2<br>Patient education and decision making	Step 3<br>Pre-transplant work-up	Step 4<br>On waiting list

Some of these steps may be difficult to move through. The IMPAKT study is investigating the processes involved at each of these 'steps' on the transplant pathway. The study team will also be working with renal units and hospitals to develop strategies to improve access to kidney transplants.

IMPAKT is a national study that will run over a three years, and will be carried out in collaboration with major transplant hospitals and renal centres in 5 states/territories. It is funded by the National Health & Medical Research Council. The major research activities under IMPAKT include:

- interviewing over 100 Indigenous and non-Indigenous Australians currently receiving dialysis treatment;
- interviewing key renal health care staff and local health services;
- a national survey of nephrologists;
- mapping current renal patient education processes at a range of service locations;
- reviewing pre-transplant work up requirements and processes;
- a modelling study of alternative kidney allocation systems.

The IMPAKT study will seek permission to look at patients' medical records to check renal health history and transplant status. No medical tests, medicines or treatments are part of this study.

The IMPAKT study team

The IMPAKT study is led by Dr Alan Cass, a kidney specialist and researcher from the George Institute for International Health at University of Sydney. Other investigators on the study are:

Dr Paul Lawton	Royal Darwin Hospital
Dr Matthew Jose	Royal Darwin Hospital
Dr James Swao	Alice Springs Hospital
Assoc/Prof Joan Cunningham	Menzies School of Health Research (Darwin)
Dr Jeannie Devitt	CRC for Aboriginal Health (Darwin)
Ms Cilla Preece	The George Institute (Sydney)
Ms Kate Anderson	The George Institute (Sydney)

The patient and staff interviews will be undertaken by Dr Jeannie Devitt and Ms Cilla Preece of the IMPAKT investigators' team. Both are experienced in particular in working with Aboriginal and Torres Strait Islander people.

Ethical approvals for IMPAKT study

The IMPAKT study has been approved by the relevant local Ethics committee under guidelines in the National Statement of ethical conduct (2001) and associated documents. If you have further inquires or concerns regarding ethical conduct of this study you can contact:

- Central Australian Human Research Ethics Committee Ph: 08 3240 7737
- HREC of NT Department of Health & Community Services and Menzies School of Health Research Ph: 08 4050 6236

Feedback from IMPAKT study

The IMPAKT study will keep people up to date through newsletters and seminars. A final report will be distributed to all participating hospitals, relevant government departments and local health services. An account of the complete research proposal is available with this information sheet if required.

Need more information? Want to make a comment?

Please contact: Dr Alan Cass or Ms Kate Anderson

Ph: 02 9993 4574 or kanderson@thegeorgeinstitute.org
