# Supplementary material for: Study Protocol – Improving Access to Kidney Transplants (IMPAKT): A detailed account of a qualitative study investigating barriers to transplant for Australian Indigenous people with end-stage kidney disease
Source: BMC Health Serv Res. 2008 Feb 4;8:31. doi: 10.1186/1472-6963-8-31 (PMC2275237; doi:10.1186/1472-6963-8-31)
Supplement: Additional file 5 — PDF, IMPAKT Project Information Sheet – patients; Project explanation provided to and discussed with patients, carers, families. [file 1472-6963-8-31-S5.pdf]

We invite you to participate in the IMPAKT study

The George Institute
ABN 90 085 953 331

Level 10 King George V Building
Royal Prince Alfred Hospital
Missenden Road Camperdown
Sydney NSW 2050 Australia

PO Box M201 Missenden Road
Sydney NSW 2050 Australia

Telephone +61 2 9993 4500
Facsimile +61 2 9993 4501

impakt@thegeorgeinstitute.org
www.thegeorgeinstitute.org

What is the IMPAKT study about?

The study is about improving access to kidney transplants for people who need them. Transplant is a sensitive issue, and we understand some people may prefer not to have this treatment. However, we want to make it easier to get a transplant for those people who do want one.

There are many steps or 'gates' on the path to getting a new kidney - things like getting the necessary medical tests done, getting enough information so you can decide what is right for you, getting your name put on the waiting list (if that is what you want), staying in good health while waiting for a kidney and so on. Some of these gates may be difficult for some patients to get through. The study will try to understand these steps or 'gates' on the pathway to a transplant.

Why are we doing this study?

In the final stages of kidney disease, the only treatments are dialysis or getting a new kidney by transplant. The best treatment for many people is a transplant. Aboriginal people and Torres Strait Islanders get kidney disease much more than other Australians but they are less likely to get a new kidney than other Australian kidney patients.

This study hopes to improve access to kidney transplants for all suitable kidney patients.

Who are the people doing the IMPAKT project?

The IMPAKT study is being led by Dr Alan Cass, a kidney specialist and researcher from the George Institute for International Health at the University of Sydney. Other people working on the study are:

Dr Paul Lawton	Royal Darwin Hospital
Dr Matthew Jose	Royal Darwin Hospital
Dr James Swao	Alice Springs Hospital
Assoc/Prof Joan Cunningham	Menzies School of Health Research (Darwin)
Dr Jeannie Devitt	CRC for Aboriginal Health (Darwin)
Ms Cilla Preece	The George Institute (Sydney)
Ms Kate Anderson	The George Institute (Sydney)

How will we do the study?

We will do the study by speaking with kidney patients, kidney specialists, staff at kidney units and local health services in five states/territories of Australia.

What will it mean if I take part in this study?

A study team member would talk to you about your kidney health experience and your opinions about education and treatments. Some people may experience some sad memories as a result.

We will also ask your permission to look at your medical records to check your kidney health history, including kidney treatment, test results and things like that. No medical tests, medicines or treatments are part of this study. No money will be paid to you if you decide to participate in the study.

What benefits can come from this study?

The IMPAKT study will benefit patients receiving dialysis treatment by improving access to kidney transplants. The study will also give a better understanding of the steps and 'gates' on the pathway to a kidney transplant.

Will I be told about the study results?

We will keep people up to date with the study through a newsletter. If you wish we will give you a copy of your interview and a final study report.

Does the IMPAKT study have ethics approval?

The project has been approved by a relevant local Ethics committee under the national guidelines. If you have any questions or concerns about the ethical conduct of this study you can contact:

- Central Australian Human Research Ethics Committee Ph: 08 3240 7737
- HREC of NT Department of Health & Community Services and Menzies School of Health Research Ph: 08 4050 6236

Need more information? Want to make a comment?

Please contact: Dr Alan Cass or Ms Kate Anderson

Ph: 02-9993 4574 or kanderson@thegeorgeinstitute.org
