# Supplementary material for: Study Protocol – Improving Access to Kidney Transplants (IMPAKT): A detailed account of a qualitative study investigating barriers to transplant for Australian Indigenous people with end-stage kidney disease
Source: BMC Health Serv Res. 2008 Feb 4;8:31. doi: 10.1186/1472-6963-8-31 (PMC2275237; doi:10.1186/1472-6963-8-31)
Supplement: Additional file 6 — PDF, IMPAKT Consent Form; Proforma for those agreeing to participate includes various conditions. [file 1472-6963-8-31-S6.pdf]

Participant Consent Form

The George Institute
ABN 90 085 953 331

Level 10 King George V Building
Royal Prince Alfred Hospital
Missenden Road Camperdown
Sydney NSW 2050 Australia

PO Box M201 Missenden Road
Sydney NSW 2050 Australia

Telephone +61 2 9993 4500
Facsimile +61 2 9993 4501

impakt@thegeorgeinstitute.org
www.thegeorgeinstitute.org

Before you sign this form please be sure that you understand what it means to be part of the study. Please read (or have read to you) the Information Sheet. Please ask the study team member to answer any questions you have.

It is important to understand:

- You do not have to take part in this study if you do not wish to.
- You can stop taking part at any time.
- You may decide not to take part in the study. This will not affect your treatment or health care.
- Information you give will be used only for this study. It will be stored in a secure place. Only study team members will have access.
- Your name and details will not be made public. Nothing written in reports will link you personally to the study.

1.	I have a copy of the study information sheet	□ Yes	□ No
2.	I agree to take part in an interview for the study	□ Yes	□ No
3.	I agree that the study team can access my kidney health records for this study	□ Yes	□ No

Participant's name:______________________________________

Signed: ____________________________ Date: ____________________

I have explained the nature and purpose of this study to the above participant and have answered their questions.

Investigator name: ______________________________________

Signed: ____________________________ Date: ____________________

Permissions for interview information

1. Do you agree that the interview be taped?	□ Yes	□ No
2. Would you like a (written) copy of your interview?	□ Yes	□ No
3. Do you wish to check the interview?	□ Yes	□ No
4. Do you agree that some of your words (not your name) be used in the study reports?	□ Yes	□ No
5. Do you want a copy of the main final report? (Include contact details below)	□ Yes	□ No
6. At the end of the study do you wish your interview record to be destroyed?	□ Yes	□ No

Participant's name:______________________________________

Signed: _____________________________ Date: ____________________

I have explained the nature and purpose of this study to the above participant and have answered their questions.

Investigator name: ______________________________________

Signed: _____________________________ Date: ____________________

Contact details:
