# Supplementary material for: Study Protocol – Improving Access to Kidney Transplants (IMPAKT): A detailed account of a qualitative study investigating barriers to transplant for Australian Indigenous people with end-stage kidney disease
Source: BMC Health Serv Res. 2008 Feb 4;8:31. doi: 10.1186/1472-6963-8-31 (PMC2275237; doi:10.1186/1472-6963-8-31)
Supplement: Additional file 7 — PDF, IMPAKT Renal nursing staff interview (IMP Q3); Questions put to renal nurses in dialysis units. [file 1472-6963-8-31-S7.pdf]

STAFF INTERVIEW
IMPQ3

Points to make:

- interested in all patients and interested in Aboriginal/Torres Strait Islander patients

Introductory Comment

Recalling what the IMPAKT study is about, is there anything in particular you'd like to say before we go through these questions?

Information and Communication

1) Could you describe your role in educating patients about their treatment options?
2) In your view does the service currently provide effective education to its renal patients?
3) In your view does the service currently provide appropriate information to its renal patients?
4) What materials or other resources are available in this unit/hospital to support you in providing patient education?
5) How do you determine whether or not a patient is understanding your communication with them?
6) What options do you have if you are concerned that the patient is not understanding
7) Have you ever worked with an interpreter with a patient?
8) In your view, are all patients here well informed about their illness and treatment options?
9) Do patients ask questions about their situation/treatments? Examples?
10) What – if any - do you think are the current barriers to patients being well-informed about their illness and treatment options? Given the authority, what would you change?
11) How – if at all – does ethnicity or cultural difference influence the way you manage patients

a) *Could you describe the key issues that Aboriginal and Torres Strait Islander patients seek your assistance with.*

Treatments

12) In your experience have you noticed patterns of choice for one treatment over another among particular groups of patients (age, gender, ethnicity)
13) What is your personal view on the relative merits of available treatment options? What about for Aboriginal/Torres Strait Islander people? (PD, HD, Tx,)

14) In your experience do patients have difficulties with costs associated with any aspects of these treatments? (consider medications/transport/accommodation)
15) How – if at all - do you think treatment location (home/satellite/hospital) affects patients' access to Tx
16) In your experience here, what are the most common psycho-social effects of dialysis treatments?

b) *In your experience what sort of issues do Aboriginal/Torres Strait Islander people find most difficult about their treatments?*
c) *Are you aware of any particular cultural issues that come up for patients needing these treatments?*

Transplant

17) Could you describe your role –if any - in preparing a patient to get on Tx list?
18) Are you aware if individual patients are on the Tx waiting list?
19) Do patients ask you about Tx? Are some patients more likely than others to ask?
20) What kinds of reasons do people give you for either being keen on or against the idea of Tx? Aboriginal/TI people?
21) Do you actively promote Tx to the patients in your care?
22) In general , do you think this site/unit actively promotes Tx as a treatment option?
23) Do you raise the issue of LRD with patients? With Aboriginal/TI patients?
24) What –if any - do you think are the particular barriers to patients from this unit/site getting a Tx? To Aboriginal/TI patients? Given the chance, what would you change?

d) *Do you have a role in discussions about individual patient suitability for Tx?*
e) *Are you personally comfortable discussing (non-clinical) aspects of transplantation with patients and families?*
f) *Are there any particular issues that might make it difficult or even impossible for you to provide information on transplantation to Aboriginal or Torres Strait Islander patients and their families?*
g) *Do you prefer to be involved or not involved in that decision-making? Why is that?*
h) *Have any Aboriginal or Torres Strait Islander patients raised the issue of a LRD with you?*
i) *What sort of understanding do you think there is in your own community about transplantation (what means, how get kidney etc).*

Compliance

25) Would you say that compliance with treatments is an issue for any particular groups of patients here (ethnicity, age, gender) ?
26) **How do you manage/respond to a patient that is regularly non-compliant e.g missing dialysis treatments?

27) In your experience here, what sort of circumstances lead to patients being non-compliant? What about Aboriginal/TI patients?
28) How – if at all - is a patient's pattern of compliance/non-compliance documented?
29) Is there an agreed review process for patients who have difficulty with compliance?
30) As far as you are aware, how does compliance impact on Tx potential?

j) ***Would you usually participate in managing Aboriginal/Torres Strait Islander patients who regularly misses dialysis treatments, medications or appointments?*
k) *What sort of strategies do you try (or recommend)?*

System & context

31) Does this dept/institution/unit have policies on social/cultural diversity?
32) Does it have policies addressing equity issues?
33) Does it have policies on interpreter use?
34) Would you say these policies are reflected in service delivery?
35) Do you think this unit/department deals fairly with clients? With Aboriginal/Torres Strait Islander clients ?
36) Do you think this unit/department deals fairly with staff? With Aboriginal/Torres Strait Islander staff?
37) What has been your main source of information in dealing with Aboriginal/Torres Strait Islander clients ?
