# Supplementary material for: Study Protocol – Improving Access to Kidney Transplants (IMPAKT): A detailed account of a qualitative study investigating barriers to transplant for Australian Indigenous people with end-stage kidney disease
Source: BMC Health Serv Res. 2008 Feb 4;8:31. doi: 10.1186/1472-6963-8-31 (PMC2275237; doi:10.1186/1472-6963-8-31)
Supplement: Additional file 8 — PFD, IMPAKT: Social support staff interview (IMP Q6); Questions put to staff involved in social support for kidney patients. [file 1472-6963-8-31-S8.pdf]

SOCIAL SUPPORT STAFF INTERVIEW
IMPQ6

Introductory comments

1) Recalling what the IMPAKT study is about, is there anything in particular you'd like to say before we go through these questions?

Information and Communication

2) Could you describe the key issues that patients seek your assistance with? Aboriginal and Torres Strait Islander patients?.
3) In your view, are patients here well informed about their illness and treatment options?
4) How do you determine whether or not a patient is understanding your communication with them?
5) What options do you have if you are concerned that the patient is not understanding you sufficiently?
6) Have you ever worked with an interpreter with a patient?

Social dimensions of treatments

7) In your experience have you noticed patterns of choice for one treatment over another among particular groups of patients (age, gender, ethnicity)
8) In terms of social or psycho-social adjustments, what is your personal view on the relative merits of available treatment options? For Aboriginal/Torres Strait Islander people? (e.g. PD, HD, Tx,)
9) What are the most common psycho-social responses associated with dialysis treatments? Have you noted patterns associated with particular groups of patients.
10) In your experience do patients have difficulties with costs associated with any aspects of these treatments? (consider medications/transport/accommodation)
11) How- if at all - do you think treatment location (home/satellite/hospital) affects patient access to transplant ?

a) In your experience what sort of issues do Aboriginal/Torres Strait Islander people find most difficult about their treatments?

b) Are you aware of any particular cultural issues that come up for patients needing these treatments?

Transplantation

12) Do you have a role in assessing patient suitability for Tx?
13) Are you aware if individual patients are on the transplant waiting list?
14) Could you describe your role in preparing a patient for Tx?

15) Do patients discuss the idea of Tx with you? What about Aboriginal or Torres Strait Islander patients? Are some patients more likely than others to ask? (i.e. younger, female etc)
16) Do you actively promote Tx to the patients in your care?
17) In general , do you think this site/unit actively promotes the idea of Tx to all suitable patients?
18) What kinds of reasons do Aboriginal/Torres Strait Islander people provide for being either keen on or against the idea of Tx.
19) In your experience how have Aboriginal or Torres Strait Islander people responded to and managed transplantation?
20) Have any Aboriginal or Torres Strait Islander patients raised the issue of a LRD with you?
21) What - if any – do you think are the barriers to Aboriginal/TI patients from this unit/site getting a Tx? Given the chance, what would you change?

c) Are you personally comfortable discussing (non-clinical) aspects of transplantation with patients and families?
d) Are there any particular issues that might make it difficult or even impossible for you to provide information on transplantation to Aboriginal or Torres Strait Islander patients and their families?
e) Do you prefer to be involved or not involved in that decision-making?
f) Have any Aboriginal or Torres Strait Islander patients raised the issue of a LRD with you?
g) What sort of understanding do you think there is in your own community about transplants (what means, how get kidney etc).

Compliance

22) Would you say that compliance with treatments is an issue for any particular groups of patients here (age, gender, ethnicity)?
23) What - if anything – is your involvement in managing or responding to patients that regularly misses dialysis treatments?
24) Do patients with compliance problems seek or initiate assistance from you?
25) In your experience here, what sort of circumstances lead to patient being non-compliant?
26) How – if at all - is a patient's pattern of compliance/non-compliance documented?
27) Is there an agreed review process for patients who have difficulty with compliance?
28) As far as you are aware, how does compliance impact on Tx potential?

h) In your experience, what sort of circumstances lead to Aboriginal/Torres Strait Islander patient being non-compliant?
i) Would you usually participate in managing Aboriginal/Torres Strait Islander patients who regularly misses dialysis treatments, medications or appointments?
j) What sort of strategies do you try (or recommend)?

System & context

29) Would you like to make any comments on the map of the local process involved in getting a patient to transplant from this site.
30) Does this dept/institution/unit have policies on social/cultural diversity?
31) Does it have policies on interpreter use?
32) Does it have policies addressing equity issues?
33) Would you say these policies are reflected in service delivery?
34) Do you think this unit/department deals fairly with clients? With Aboriginal/Torres Strait Islander clients ?
35) Do you think this unit/department deals fairly with staff? With Aboriginal/Torres Strait Islander staff?
36) What has been your main source of information in dealing with Aboriginal/Torres Strait Islander clients ?
