# Supplementary material for: Study Protocol – Improving Access to Kidney Transplants (IMPAKT): A detailed account of a qualitative study investigating barriers to transplant for Australian Indigenous people with end-stage kidney disease
Source: BMC Health Serv Res. 2008 Feb 4;8:31. doi: 10.1186/1472-6963-8-31 (PMC2275237; doi:10.1186/1472-6963-8-31)
Supplement: Additional file 9 — PDF, IMPAKT Nephrologist interview (IMP Q5); Questions put to nephrologists. [file 1472-6963-8-31-S9.pdf]

NEPHROLOGIST INTERVIEW
IMPQ5

Individual Comment

1) Recalling what the IMPAKT study is about, is there anything in particular you'd like say about the issue of access to Tx in general?

Treatment choices

2) In your experience have you noticed patterns of choice for one treatment over another other among particular groups of patients (age, gender, ethnicity)
3) Aside from medical suitability, what limitations - if any, (including costs) - are there on patient choice between various treatment options?
4) What is your personal view on the relative merits of available treatment options. What about for Aboriginal/Torres Strait Islander people? (PD, HD, Tx,)
5) How - if at all – do you think treatment location (home/satellite/hospital) affects a patients' likelihood of Tx.
6) Once a patient has begun on a particular treatment modality what is the regularity of review at this site, and how is this documented?
7) How- if at all – do you think a patient's social circumstances impact on the effectiveness of treatments?

Assessing suitability for transplant

8) How do you determine the appropriate time to introduce the idea of transplantation to patients
9) Could you describe the process at this site to evaluate medical suitability for Tx ? [distinguish between sites as necessary]
10) Could you describe the process at this site to evaluate psychosocial suitability for Tx? [distinguish between sites as necessary]
11) Which staff have a formal role in assessment processes?
12) How – if at all - do these various assessment processes account for patients with limited English and/or different cultural backgrounds?
13) Do you advise patients that they are not suitable for Tx? Under what circumstances would you change that position?
14) What are the decision-making processes at this site in regard to patients interested in Tx but considered as 'marginal' or 'controversial' in terms of suitability (i.e. where there is uncertainty or disagreement as to suitability for Tx)?
15) What -for you personally - are the key considerations when assessing such a patient?
16) What – if any - are the review cycles or processes for potentially appropriate Tx patients (i.e. those who may be either marginally suitable or undecided?)

Transplant

17) In your experience what key issues/concerns arise for patients considering transplant? What about Aboriginal or Torres Strait Islanders patients?

18) Do you actively promote Tx to all suitable patients in your care?
19) In general , does the site/units you work at actively promote Tx as a treatment? [distinguish between sites as necessary]
20) Do patients ask you about Tx? Are some patients more likely than others to ask?
21) How – if at all – would you present life expectancy data to prospective Tx patients?
22) Do you think there are particular barriers to patients you manage at this unit/site getting a Tx? How might that be changed? [distinguish between sites as necessary]
23) Who has the responsibility to communicate with patients about their current Tx status (including periods of being on/off the list)
24) How is waiting time accrued?
25) In general, what have been the outcomes for Aboriginal/Torres Strait Islander patients from this site [distinguish between sites as necessary] who obtain a Tx (i.e. are they better, worse, or equal to national outcomes) & what do you think the reasons for that are?

Compliance

26) Could you comment on what you understand by 'compliance' and describe how you assess it in relation to dialysis patients.
27) Would you say that compliance with treatments is an issue for particular groups (age, gender, ethnicity) of patients that you manage here? [distinguish between sites as necessary]
28) In your experience here, what sorts of circumstances lead to a patient being non-compliant?
29) How – if at all - is a patient's pattern of compliance/non-compliance documented?
30) What – if any - is your role in managing/responding to patients that are regularly non-compliant e.g. missing dialysis treatments?
31) Is there an agreed review process for patients who have difficulty with compliance?
32) How does compliance (or lack of compliance) impact on Tx potential?

Information and Communication

33) Could you describe briefly your role in advising and educating patients about their treatment options?
34) In your view do the units/departments you currently deal with provide effective education to renal patients. [distinguish between sites as necessary]
35) How do you determine whether or not a patient is understanding your communication with them?
36) What options do you have if you are concerned that the patient is not understanding you sufficiently?
37) Have you ever worked with an interpreter with a patient?
38) In your view, are the patients you manage generally well informed about their illness and treatment options?
39) What – if any - are the barriers to patients you manage being well-informed about their illness and treatment options? Given an opportunity, what would you change?
40) How – if at all - does ethnicity or cultural difference influence the way you manage patients?

System & context

41) Does this dept/institution/unit have policies on social/cultural diversity?
42) Does it have policies on interpreter use?

43) Does it have policies addressing equity issues?
44) Would you say service delivery currently reflects these policies?
45) Do you think this unit/department deals fairly with clients? With Aboriginal/Torres Strait Islander clients ?
46) Do you think this unit/department deals fairly with staff? With Aboriginal/Torres Strait Islander staff?
47) What has been your main source of information in dealing with Aboriginal/Torres Strait Islander clients ?
