# Supplementary material for: Study Protocol – Improving Access to Kidney Transplants (IMPAKT): A detailed account of a qualitative study investigating barriers to transplant for Australian Indigenous people with end-stage kidney disease
Source: BMC Health Serv Res. 2008 Feb 4;8:31. doi: 10.1186/1472-6963-8-31 (PMC2275237; doi:10.1186/1472-6963-8-31)
Supplement: Additional file 10 — PDF, IMPAKT Patient Interview – full version (IMP Q4); Questions put to patients (as appropriate). [file 1472-6963-8-31-S10.pdf]

PATIENT INTERVIEW
IMPQ4

Introductions

1) How are you feeling today?

Health history
2) Can you tell about me what happened to your kidneys?
3) What do you think is the cause of your kidney disease?
4) What have the specialists told you was the cause of your kidney problem?
5) Does anyone else in your family have this same kidney problem?
6) Are you taking medications/medicines as well - can you tell me what they are?
7) Do you have to do any other particular things to do as part of your treatment (diet, fluid)
8) Do you have a local doctor/GP here as well as the kidney specialist ?

Social & Psychosocial context
9) Who is your main helper/support person/carer?
10) How do they help you?
11) Do you talk to your family about this treatment? What do they say?
12) What are the main problems for you at the moment ?
13) Where do you go for help?

Attitudes/values
14) How has your life changed since you started on dialysis/got a Tx?
15) How do you feel now about your situation? (anger, sadness, OK, shame, disappointment)
16) Do you think anyone is to blame for this illness?
17) Is there anything you could do to improve your own situation? How would you do that?
18) What's most important to you at the moment?

Treatments
19) How long have you been on this treatment?
20) Are you happy about the treatment you are on at the moment?
21) Why did you choose that one?
22) Do you think you will be staying on this treatment?
23) Are you involved in decisions about your treatment?

24) How do you feel when you go on dialysis/the dialysis machine?
25) Does dialysis cause you any sort of problems - can you tell me about them?
26) Do you ever miss out your dialysis treatments? What sort of things cause you to miss out dialysis?
27) What does missing dialysis do to your health?

Information & Communication
28) How do you learn about your kidney illness and the different treatments?
29) Do you get enough information to help you understand things well?
30) Is there anything you would really like to know more about?

31) Do you have any problems understanding what the kidney specialist talks to you about? What about the nurses?
32) Have you said anything to them about that problem?
33) Have you ever asked the specialist or nurses questions about your illness or your treatment? What questions were they?
34) What sort of things have other patients told you about the treatments?
35) (For non-English/ESL) Are you able to get any information in your own language?
36) Have you ever had an interpreter to help you?

Transplantation

Group A: Patients on Dialysis

1) Have you thought about a Tx?
2) Could you talk about why you're interested/not interested in a Tx?
3) Have the specialists talked to you about getting a Tx? When was that?
4) Has anyone else talked to you about getting a Tx?
5) Have you ever asked anyone about getting a Tx?
6) Are you on the Tx list at the moment?
7) (If 'yes') What are the main things that you need to do if you want to get a Tx?
   (If 'no') What are the main things that you need to do if you want to get on the list?
8) Do you know anyone who has had a Tx? What do they say?
9) Have you heard any good/bad stories about other people who had a Tx? Does that make you think that Tx is good/bad?
10) From what you know, do you think people who get a Tx are happier?
11) Does your family know about transplantation? What do they say about it?
12) Have you heard about family members giving a kidney? What do you think?

Group B: Current Tx patients

1) How has Tx changed things for you? Better/not better?
2) How did you make your decision about getting another kidney?
3) What was the most difficult thing for you as far as having a Tx?
4) Does your family know about your transplant? What do they say about it?
5) As a person with a Tx, what are the main things that you need to do for your health?
6) Did you know anyone else who had a Tx? Did you talk with them about it first?
7) Had you heard any good/bad stories about other people who had a Tx?
8) Do you have any problems or worries now about this treatment?

Group C: Patients who have had Tx/s and then moved back to dialysis

1) Do you mind describing what happened with the Tx?
2) Do you think you would say 'yes' again if another kidney came up for you?
3) What was the most difficult thing for you as far as having a Tx?
4) Did you know anyone else who had a Tx? Did you talk with them about it first?
5) Had you heard any good/bad stories about other people who had a Tx?

F. Satisfaction

38 & 39 see Record of Interview sheet.

40) When did you last talk with the kidney specialist?
41) Do the staff here treat you well?
42) Is this a friendly and comfortable place to have your dialysis treatment?
43) If you were in charge of this unit/dept is there any particular things you would change?
