# Supplementary material for: Study Protocol – Improving Access to Kidney Transplants (IMPAKT): A detailed account of a qualitative study investigating barriers to transplant for Australian Indigenous people with end-stage kidney disease
Source: BMC Health Serv Res. 2008 Feb 4;8:31. doi: 10.1186/1472-6963-8-31 (PMC2275237; doi:10.1186/1472-6963-8-31)
Supplement: Additional file 11 — PDF, IMPAKT Patient Interview -outline – plain English (for patients); A version of the patient questions in more accessible language. [file 1472-6963-8-31-S11.pdf]

PATIENT INTERVIEWS OUTLINE
IMPQ4 (ESL)

Meet the interviewer

Your main points
Is there anything you would like say about your situation now?

Family and feelings
- ✓ feelings
- ✓ helpers/carers (family/friend/other)
- ✓ family discussions
- ✓ problems & assistance

Thoughts and Attitudes
- ✓ effects on life
- ✓ feelings including blame/anger
- ✓ how I might improve my situation
- ✓ important things for me at the moment

Health history
- ✓ my story & my ideas of causes
- ✓ specialist's idea
- ✓ in family?
- ✓ treatment – medicines, other things
- ✓ local doctor/GP

Treatments
- ✓ happy with treatment
- ✓ reasons for choice, involved in decision
- ✓ staying on this treatment
- ✓ how feel on treatment,
- ✓ any problems
- ✓ missing treatments/medications & effects on health

Learning and talking
- ✓ ways of learning
- ✓ enough information
- ✓ things I would like to know more about
- ✓ most important things learned
- ✓ any problems in understanding specialists or nurses
- ✓ questions
- ✓ information from other patients
- ✓ using interpreters

Transplant

For Patients on Dialysis

- ✓ interest in transplant, reasons for/against, possible benefits
- ✓ who spok to you about it
- ✓ asked anyone
- ✓ (if interested) on the list?
- ✓ other people's stories, experience - what think
- ✓ family ideas about transplant
- ✓ knowledge & ideas about living donors

For Patients with transplant

- ✓ effects on life – good/bad
- ✓ how decided
- ✓ difficulties in deciding
- ✓ family ideas
- ✓ keeping good health
- ✓ stories about other people
- ✓ any worries/problems

For Patients who had transplant/s and now on dialysis again

- ✓ effects on life – good/bad
- ✓ story of what happened to transplant
- ✓ interest in another transplant
- ✓ problems in getting another transplant
- ✓ experiences of others- stories

Satisfied with services

- ✓ good medical treatment here?
- ✓ what other help needed
- ✓ specialist – doing a good job?
- ✓ last time talked with specialist?
- ✓ staff looking after well?
- ✓ getting involved in deciding my treatment
- ✓ friendly & comfortable dialysis place
- ✓ ideas about anything to change
