# Supplementary material for: Study Protocol – Improving Access to Kidney Transplants (IMPAKT): A detailed account of a qualitative study investigating barriers to transplant for Australian Indigenous people with end-stage kidney disease
Source: BMC Health Serv Res. 2008 Feb 4;8:31. doi: 10.1186/1472-6963-8-31 (PMC2275237; doi:10.1186/1472-6963-8-31)
Supplement: Additional file 12 — PDF, IMPAKT Patient Interview – prompt points only; A version of the patient questions showing a list of prompts for interviewer. [file 1472-6963-8-31-S12.pdf]

PATIENT INTERVIEW
IMPQ4 (prompts only)

Introductions
- Interviewer introduces self, brief background (where from, family etc) why interested to talk to person.
- Emphasise: confidentiality; value in improving situation.
- Indicate topics we will be talking about.

Key concerns of interviewee
Is there anything in particular you'd like say about your current situation as a dialysis/Tx patient/person ?

Social & Psychosocial context
Can you tell me a little bit about yourself firstly?

- ✓ feelings
- ✓ helpers/carers (family/friend/other)
- ✓ family discussions
- ✓ problems & assistance

Is there anything else you want to say about that before we go on?

Attitudes/values

- ✓ effects on life
- ✓ feelings including blame/anger
- ✓ actions to improve situation
- ✓ current priorities

Health history

- ✓ your story & ideas of causes
- ✓ specialist's idea
- ✓ in family?
- ✓ treatment – medicines, other things
- ✓ local doctor/GP

Treatments
- ✓ happy with treatment
- ✓ reasons for choice, involvement in decision
- ✓ staying on this treatment
- ✓ feeling on treatment,
- ✓ problems
- ✓ missing treatments/medications & effects on health

Information & Communication

- ✓ ways of learning
- ✓ enough information
- ✓ things like to know more about
- ✓ most important things that learned
- ✓ problems in understanding a) specialists b) nurses
- ✓ questions
- ✓ information from other patients
- ✓ other languages/interpreters

Transplant

Group A: Patients on Dialysis
- ✓ interest in Tx, reasons for, against, possible benefits
- ✓ who spoken to you about it
- ✓ asked anyone
- ✓ (if interested) on the list?
- ✓ preparations
- ✓ other people's stories, experience - what think
- ✓ family knowledge & views of Tx
- ✓ knowledge & views of LRD

Group B: Current Tx patients
- ✓ effects on life – positive/negative
- ✓ how decided
- ✓ difficulties in deciding
- ✓ family knowledge & views
- ✓ maintaining good health
- ✓ experiences of others - stories
- ✓ any current worries/problems

Group C: Patients who have had Tx/s and then moved back to dialysis
- ✓ story of what happened to Tx
- ✓ interest in another Tx
- ✓ difficulties in Tx process
- ✓ experiences of others- stories

Satisfaction

- ✓ standard of medical care
- ✓ what other help
- ✓ specialist – doing a good job?
- ✓ last consultation with specialist?
- ✓ staff treatment
- ✓ participation in treatment decisions
- ✓ friendly & comfortable dialysis place
- ✓ If you were in charge of this unit/dept is there any particular things you would change?
