# Supplementary figures and images for: Study Protocol – Improving Access to Kidney Transplants (IMPAKT): A detailed account of a qualitative study investigating barriers to transplant for Australian Indigenous people with end-stage kidney disease
Source: BMC Health Serv Res. 2008 Feb 4;8:31. doi: 10.1186/1472-6963-8-31 (PMC2275237; doi:10.1186/1472-6963-8-31)

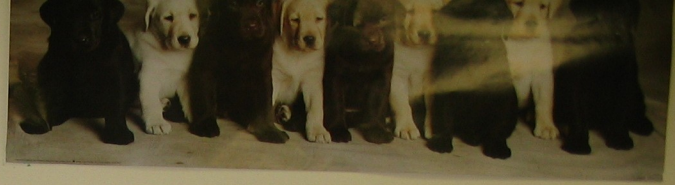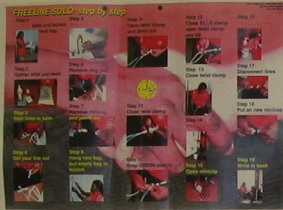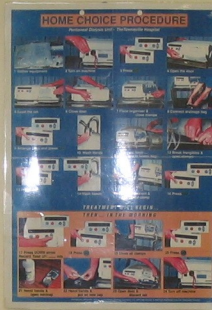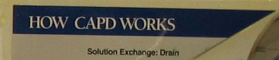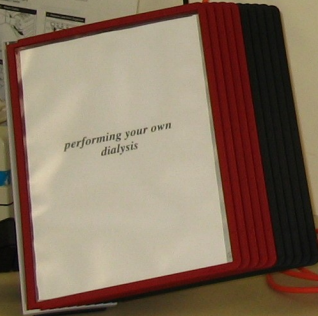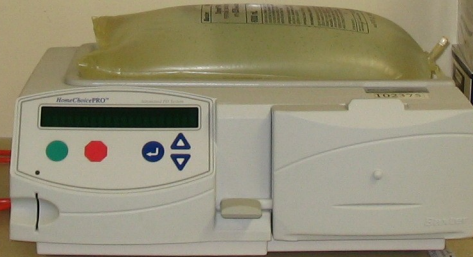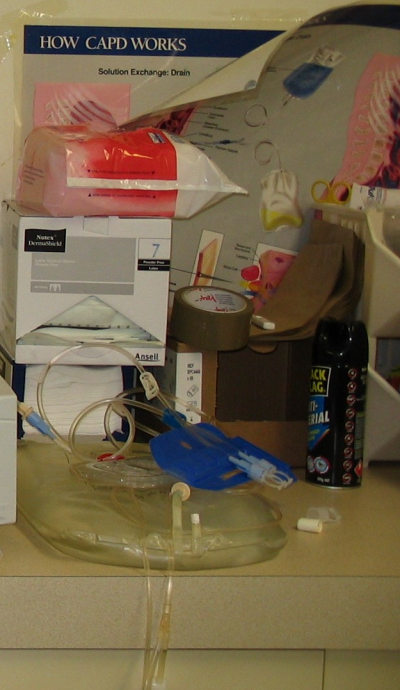

Supplement: Additional file 13 — PDF, Peritoneal Dialysis cycler machine; Photo of peritoneal cycler machine in a home therapies instruction setting. [file 1472-6963-8-31-S13.pdf]

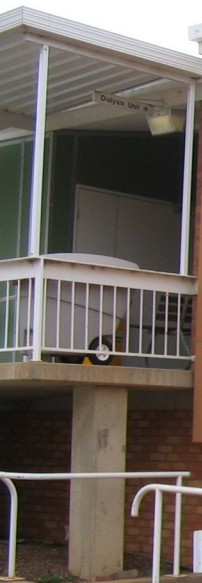

Dialysis Unit  
← Entrance via Ramp

Dialysis Unit

DO NOT REMOVE  
THIS SIGN

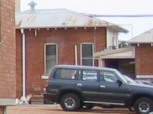

Supplement: Additional file 14 — PDF, A regional hospital based dialysis unit; Photo. [file 1472-6963-8-31-S14.pdf]

# **Kimberley Satellite Dialysis Centre**

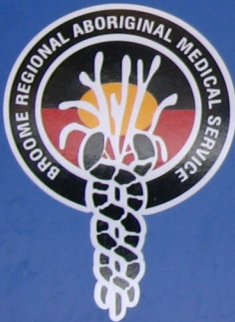

**BROOME REGIONAL  
ABORIGINAL  
MEDICAL SERVICE**

Supplement: Additional file 15 — PDF, Remote area Aboriginal Community Controlled Dialysis Unit; Photo of plaque explaining purpose of facility. [file 1472-6963-8-31-S15.pdf]

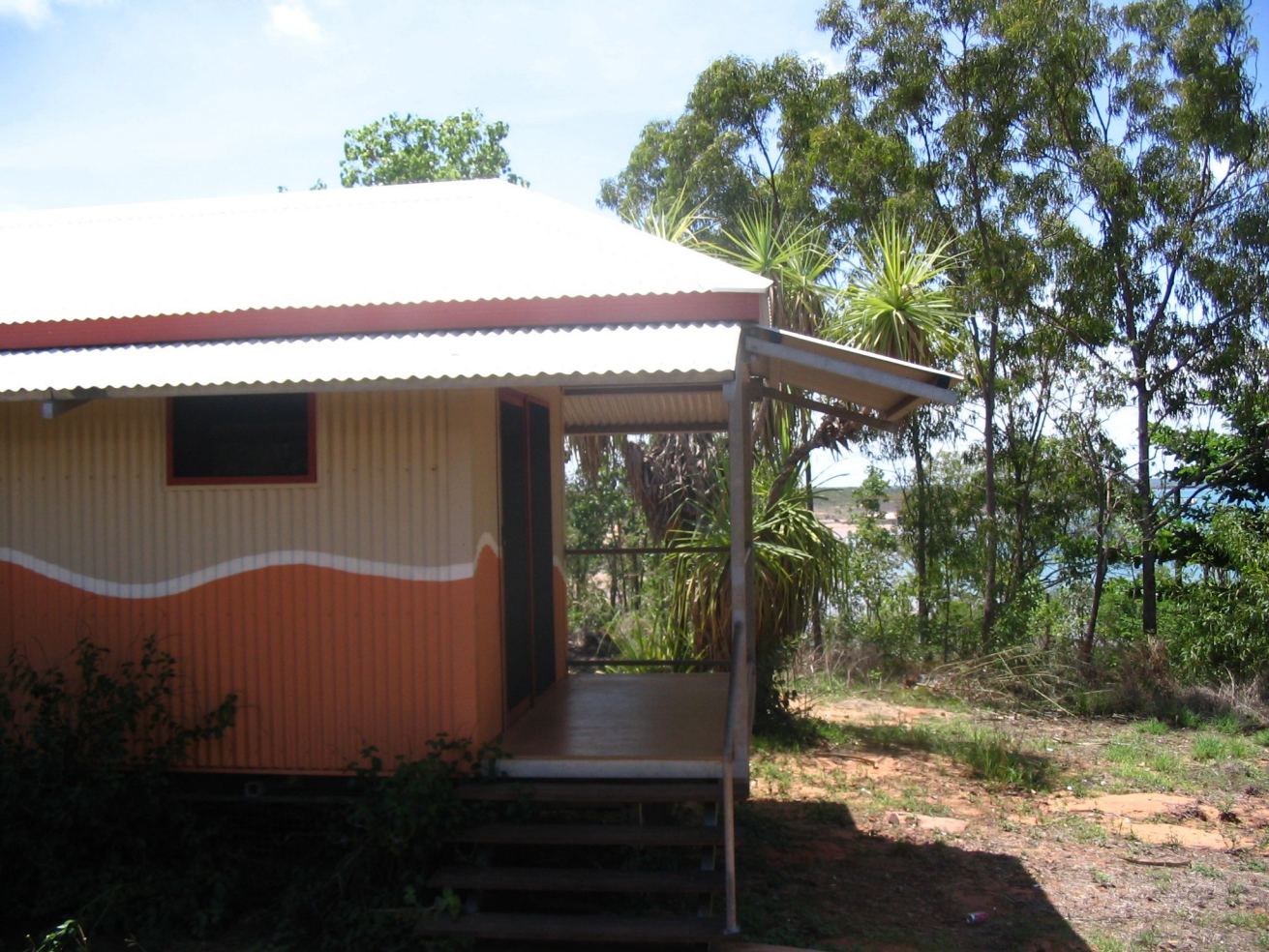

Supplement: Additional file 16 — PDF, Remote area Indigenous home haemodialysis facility; Photo. [file 1472-6963-8-31-S16.pdf]

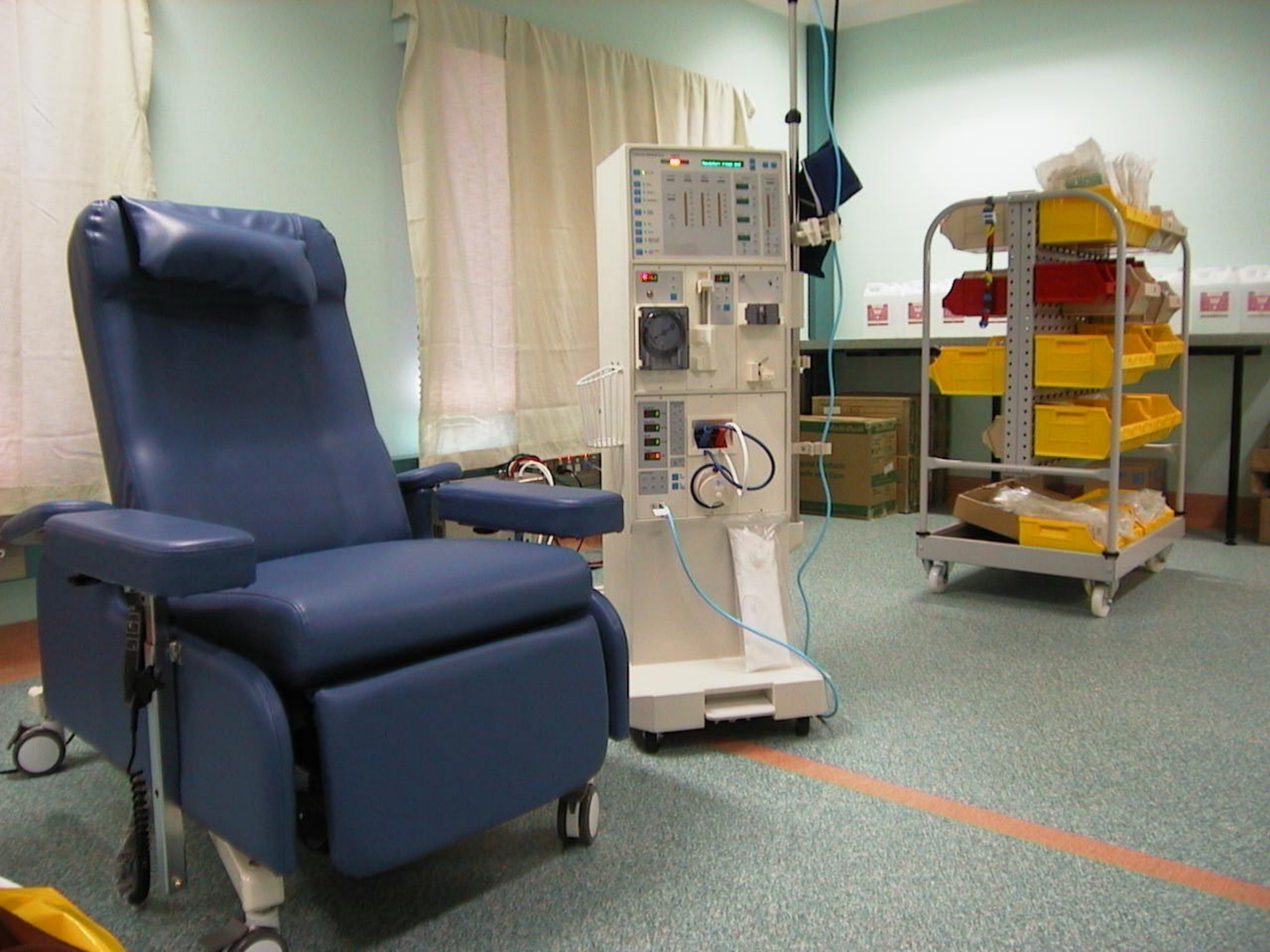

Supplement: Additional file 17 — PDF, Dialysis chair and haemodialysis machine; Photograph. [file 1472-6963-8-31-S17.pdf]

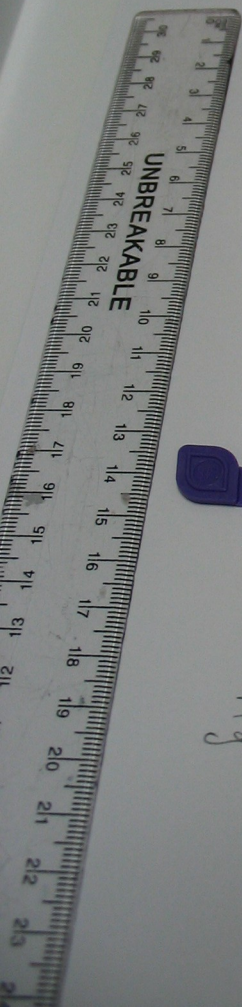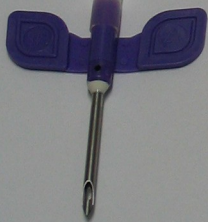

14g

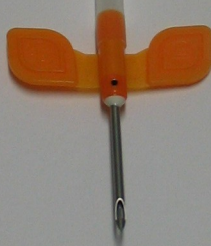

15g

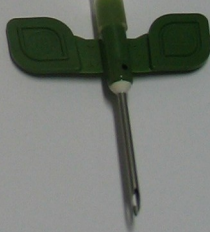

16g

Supplement: Additional file 18 — PDF, Dialysis needles; Photo – needles that are inserted into fistula at every dialysis session. [file 1472-6963-8-31-S18.pdf]

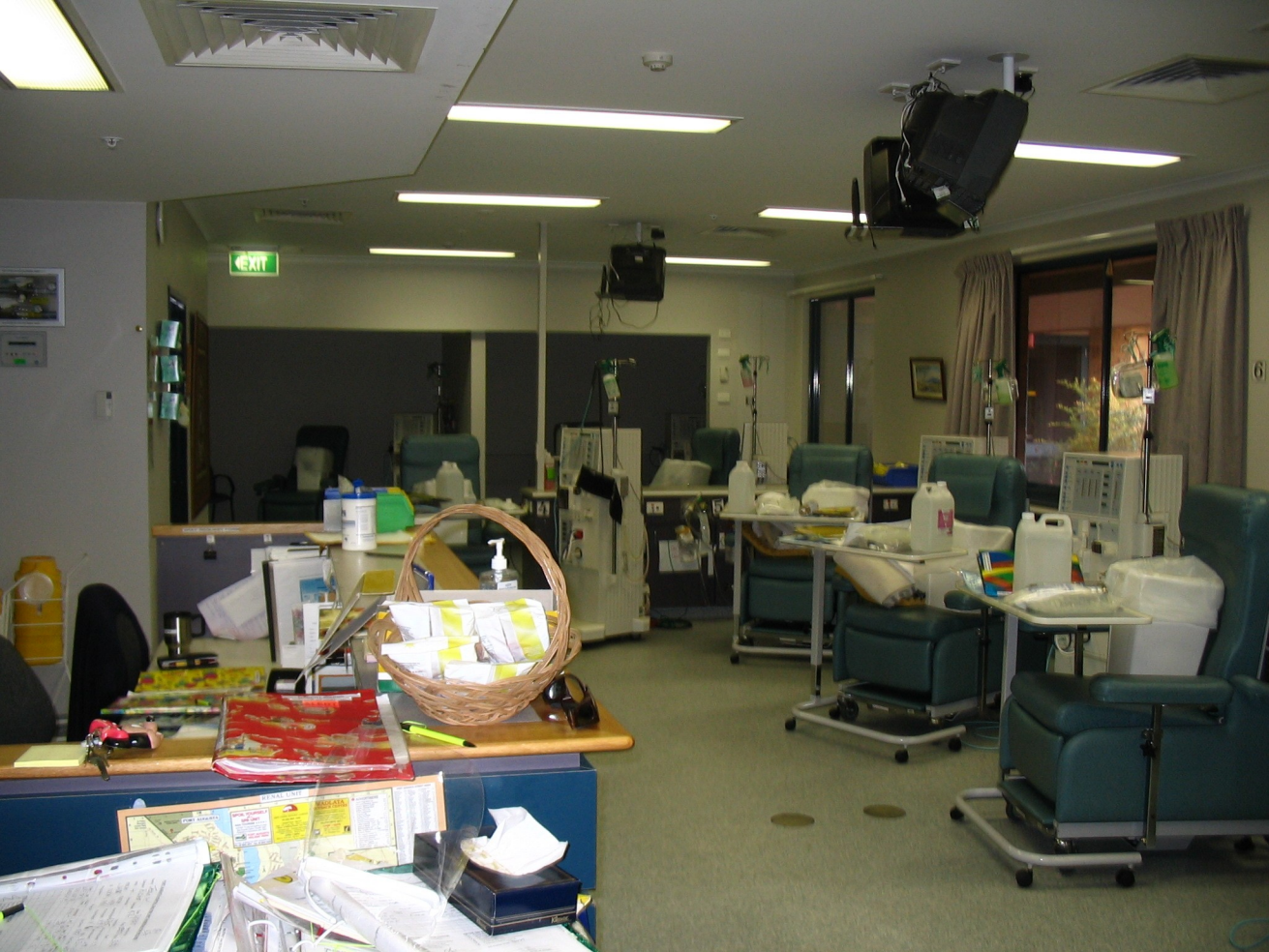

Supplement: Additional file 19 — PDF, Small dialysis unit following treatment sessions; Photo. [file 1472-6963-8-31-S19.pdf]

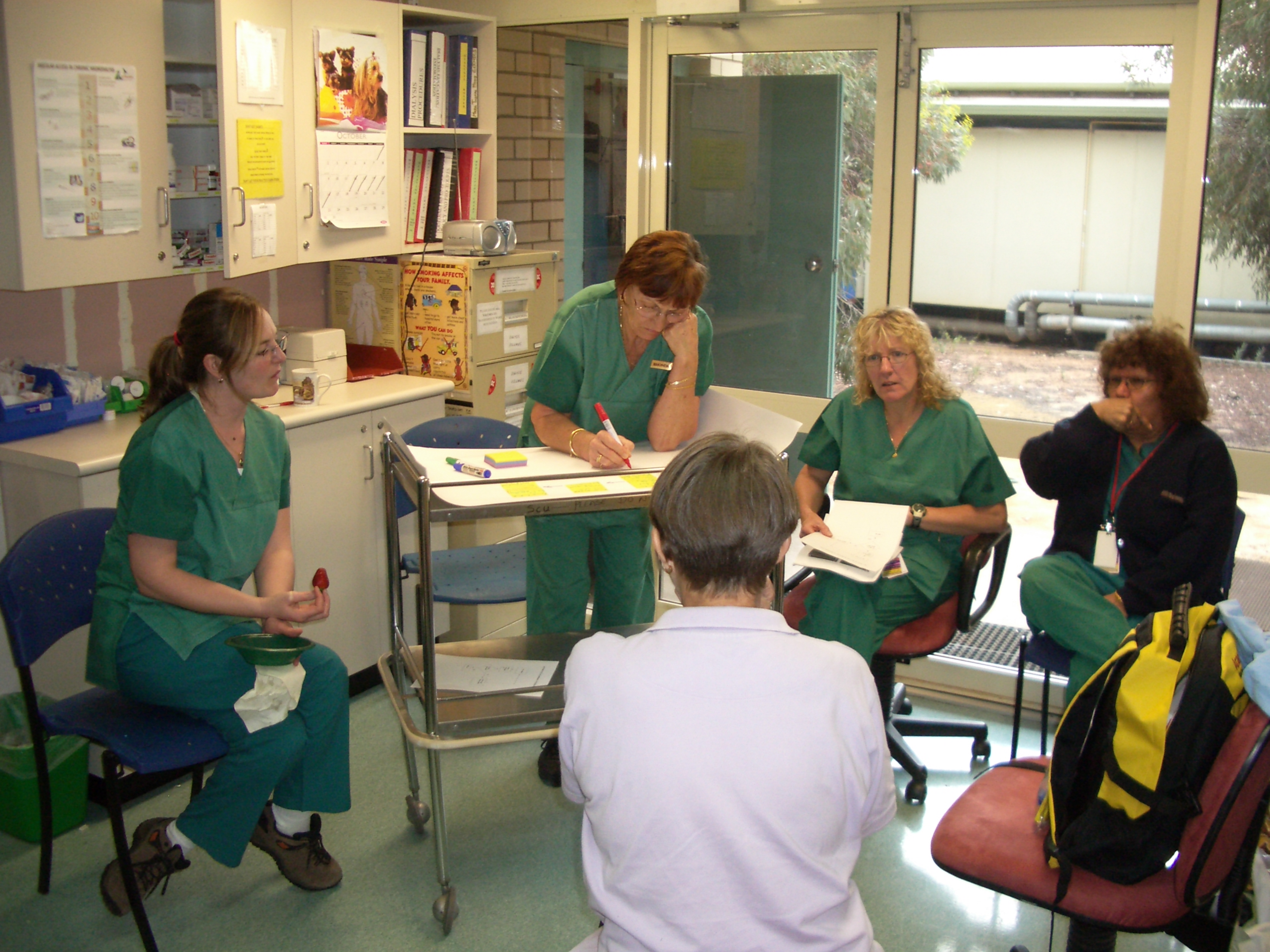

Supplement: Additional file 23 — PDF, Renal staff working on a 'patient journey' process map; Photo. [file 1472-6963-8-31-S23.pdf]

17/10/05

# RENAL UNIT PROCESS MAP: IMPAKT

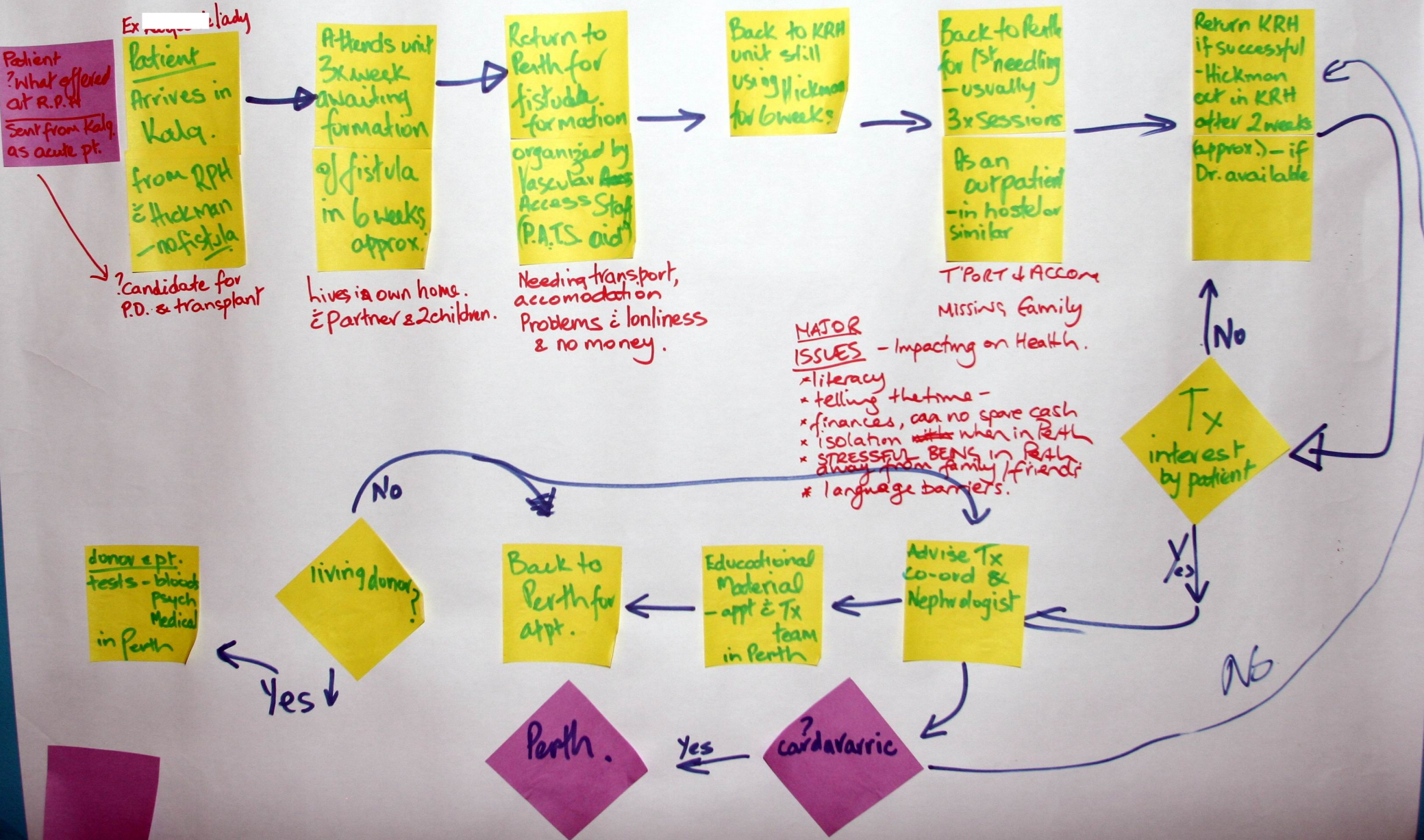

Supplement: Additional file 24 — PDF, Completed Original version of 'patient journey' process map; Photo. [file 1472-6963-8-31-S24.pdf]
