# Supplementary material for: Study Protocol – Improving Access to Kidney Transplants (IMPAKT): A detailed account of a qualitative study investigating barriers to transplant for Australian Indigenous people with end-stage kidney disease
Source: BMC Health Serv Res. 2008 Feb 4;8:31. doi: 10.1186/1472-6963-8-31 (PMC2275237; doi:10.1186/1472-6963-8-31)
Supplement: Additional file 20 — PDF, IMPAKT Record of Interview – Patient; Details of interview sociodemographic data of interviewee. [file 1472-6963-8-31-S20.pdf]

RECORD OF INTERVIEW

ID Number…………

DATE	SITE	CATEGORY	I/ER
/ / 05		Patient/family/other	CP/ JD

Satisfaction

38) Do you think the medical treatment and care here is……

**v.good**	**good**	**OK**	**not very good**

39) Do you think your specialist is doing a good job for you? Would you say its…

**v.good**	**good**	**OK**	**not very good**

Socio-demographic data

Age	**20-29**	**30-39**	**40-49**	**50-59**	**60-69**	**70+**
Gender						
Ethnic affiliations/heritage						
Where living before starting dialysis						
place living now	own home		rented		hostel	
Married						
No Dependents	children/other					
Main Carer						
Other services used	e.g. paid carer, meals on wheels etc					
First language						
Other languages						
Employed now?						
Employed before dialysis?						
Education/years of school?						
Religious affiliation						
Own a car?						
Own a phone?						
Own a home computer?						
Use the internet?						
Read English?	v.well	well	OK	not v.well	no	

Card sort

A person must have lots medical tests before they get a new kidney.	NT	T	
A person waiting for a new kidney must stay fit and healthy.	NT	T	
Sometimes a new kidney stops working.<br>That person must go back to dialysis.	NT	T	
After a person gets a new kidney they must stay in hospital for a long time – up to 1 month.	NT	T	
The operation for a new kidney happens in a big city hospital in ......Brisbane/Perth/Adelaide/Sydney.	NT	T	
Later on, after the new kidney settles down and everything is working well, a person with a new kidney might go home.	NT	T	
When a person has the operation to get a new kidney sometimes things go wrong.	NT	T	
My kidneys can get better.	NT	T	
A person with a new kidney can stop taking medicines.	NT	T	
When a person gets a new kidney there will be no more blood tests and no more kidney doctor visits.	NT	T	
Missing dialysis sessions helps a person to get a new kidney.	NT	T	
When a person gets called for a new kidney all their family can go with them.	NT	T	

Notes
