# Supplementary material for: Study Protocol – Improving Access to Kidney Transplants (IMPAKT): A detailed account of a qualitative study investigating barriers to transplant for Australian Indigenous people with end-stage kidney disease
Source: BMC Health Serv Res. 2008 Feb 4;8:31. doi: 10.1186/1472-6963-8-31 (PMC2275237; doi:10.1186/1472-6963-8-31)
Supplement: Additional file 21 — PDF, IMPAKT Record of Interview – Renal Staff; Details of interview; socio-demographics of interviewee; ranking of hospital services. [file 1472-6963-8-31-S21.pdf]

# RECORD OF INTERVIEW

ID Number…………

| DATE | SITE | CATEGORY | I/ER |
|---|---|---|---|
| / / 05 | | RS /SS /NE | CP/ JD/ AC |

**Job Title……………………………………………………………………..............**

**Locations currently working at:………………………………………..............**

**Organisational culture/values**

Could you describe the extent that your work place emphasizes:

| | 1 | 2 | 3 | 4 | 5 | can't say |
|---|---|---|---|---|---|---|
| efficiency | | | | | | |
| economy | | | | | | |
| patient-centred care | | | | | | |
| shared decision-making | | | | | | |
| clinical excellence | | | | | | |
| staff development | | | | | | |
| patient development/empowerment | | | | | | |

**(1 = 'Not at all' 5 = 'very strongly')**

In your judgement, is the standard of medical care here…..

| v.good | good | adequate | less than adequate |
|---|---|---|---|

**Social-demographic**

| Age | 20-29 | 30-39 | 40-49 | 50-59 | 60-69 | 70+ |
|---|---|---|---|---|---|---|
| | | | | | | |
| Gender | | | | | | |
| Ethnic Affiliations | | | | | | |
| First language | | | | | | |
| Other languages | | | | | | |
| Time in current position | | | | | | |
| Time at site | | | | | | |
| Renal training | | | | | | |
| Cross cultural training | | | | | | |
| Training to work with interpreters | | | | | | |

**Notes**
