# Supplementary material for: Study Protocol – Improving Access to Kidney Transplants (IMPAKT): A detailed account of a qualitative study investigating barriers to transplant for Australian Indigenous people with end-stage kidney disease
Source: BMC Health Serv Res. 2008 Feb 4;8:31. doi: 10.1186/1472-6963-8-31 (PMC2275237; doi:10.1186/1472-6963-8-31)
Supplement: Additional file 22 — PDF, IMPAKT Record of Interview – Patient Educators; Details of interview, socio-demographics of interviewee; notes on education programs. [file 1472-6963-8-31-S22.pdf]

RECORD OF INTERVIEW

ID Number…………

DATE	SITE	CATEGORY	I/ER
/ / 05		EDUCATION	CP/ JD

Job Title………………………………………………………………………

Locations currently working at:………………………………………….

<u>Subjective Evaluative Comments</u> (to follow questions 1-6)

In your experience which of the following best describe the kinds of educational activities currently undertaken at this site. Circle as many of these terms as you wish.

one-to-one
face-to-face
patient groups
involves family

highly interactive
passive learners
active learners
group learning
shared learning
'two-way'

conversation-style
lecture-style

well-planned
opportunistic
targetted
personalised
ad hoc
on-the-run
uncoordinated
highly variable quality
consistent quality

friendly
authoritarian
empowering
effective
appropriate
waste of time
enjoyed by patients
enjoyed by educator/staff
difficult
interesting

multi-disciplinary
outsourced
incorporates support resources

Add any other words you think describe these activities:

………………………. ……………………………….
……………………….. ……………………………….
……………………….. ………………………………..

Organisational culture/values

Could you describe the extent that your work place emphasizes:

	1	2	3	4	5	can’t say
efficiency						
economy						
patient-centred care						
shared decision-making						
clinical excellence						
staff development						
patient development/empowerment						

(1 = ‘Not at all’ 5 = ‘very strongly’)

In your judgement, is the standard of medical care here…..

v.good	good	adequate	less than adequate

Social-demographic

Age	20-29	30-39	40-49	50-59	60-69	70+
Gender						
Ethnic Affiliations						
First language						
Other languages						
Time in current position						
Time at site						
Renal training						
Cross cultural training						
Training to work with interpreters						

Notes
