# Supplementary material for: Study Protocol – Improving Access to Kidney Transplants (IMPAKT): A detailed account of a qualitative study investigating barriers to transplant for Australian Indigenous people with end-stage kidney disease
Source: BMC Health Serv Res. 2008 Feb 4;8:31. doi: 10.1186/1472-6963-8-31 (PMC2275237; doi:10.1186/1472-6963-8-31)
Supplement: Additional file 25 — PDF, Re-drafted version of 'patient journey' process map; Photo. [file 1472-6963-8-31-S25.pdf]

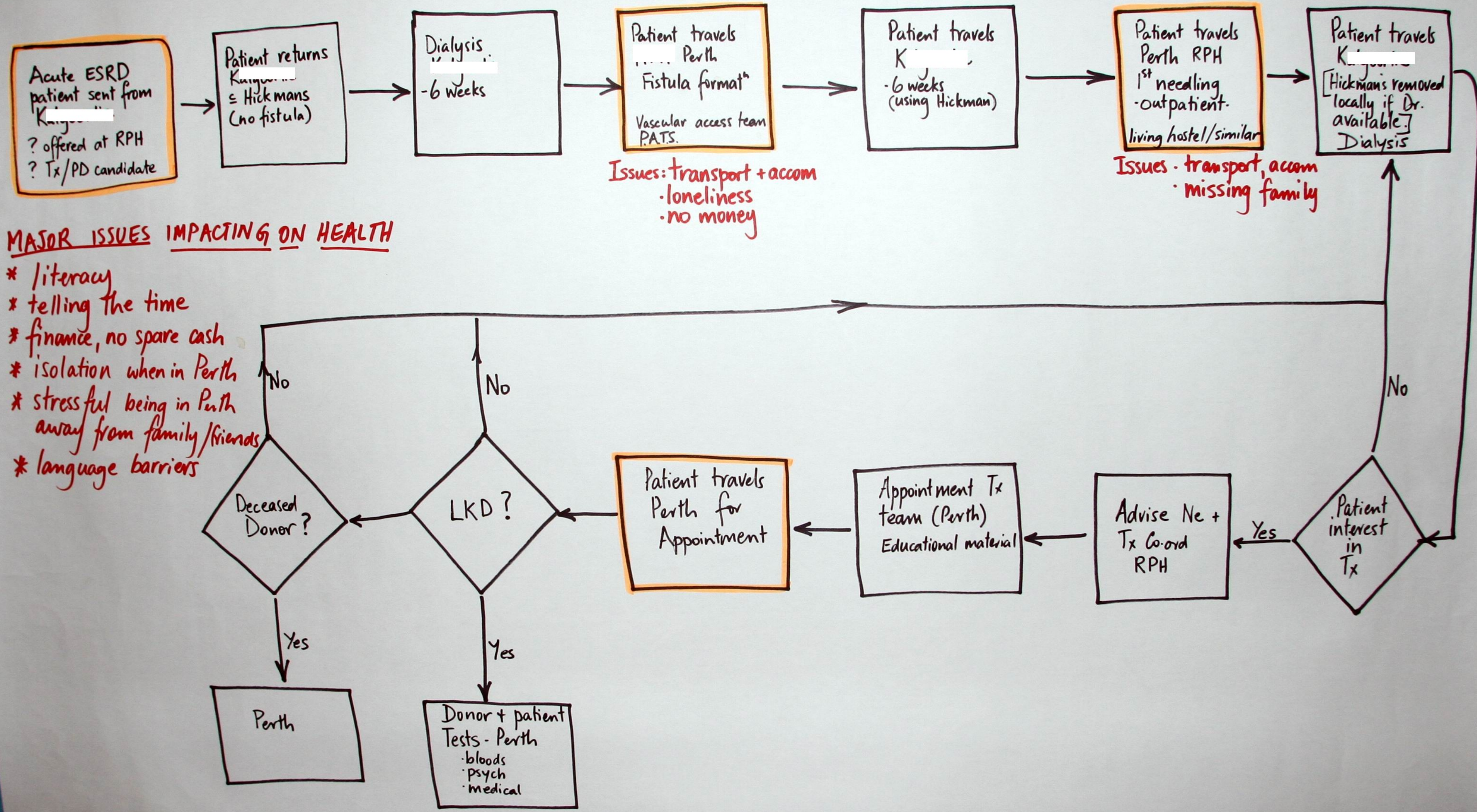

Summary of process map K[redacted] RENAL UNIT 17/10/05
