# Supplementary material for: Study Protocol – Improving Access to Kidney Transplants (IMPAKT): A detailed account of a qualitative study investigating barriers to transplant for Australian Indigenous people with end-stage kidney disease
Source: BMC Health Serv Res. 2008 Feb 4;8:31. doi: 10.1186/1472-6963-8-31 (PMC2275237; doi:10.1186/1472-6963-8-31)
Supplement: Additional file 26 — PDF, IMPAKT Questionnaire: Educational (Patient) Programs Description IMP Q1; Questions put to CKD and other patient educators. [file 1472-6963-8-31-S26.pdf]

EDUCATIONAL PROGRAMS DESCRIPTION IMPQ1	impakt Improving Access to Kidney Transplants

Information and Communication

1) Could you describe your role in educating patients about their treatment options?
2) What do you find most difficult in relation to patient education?
3) What do you think patients currently find most difficult in learning about the illness and options?
4) In your view does the service currently provide effective education to its renal patients?
5) In your view does the service currently provide appropriate information to its renal patients?
6) What materials or other resources are available in this unit/hospital to support you in providing patient education?
7) How do you determine whether or not a patient is understanding your communication with them?
8) What options do you have if you are concerned that the patient is not understanding.
9) Have you ever worked with an interpreter with a patient?
10) In your view, are all patients here well informed about their illness and treatment options?
11) Do patients ask questions about their situation/treatments? Examples?
12) How –if at all – do you think education and information processes at this site might constitute a barrier to Aboriginal/Torres Strait Islander patients accessing Tx? Given a chance, what would you change?
13) How – if at all – does ethnicity or cultural difference influence the way you manage patients

PART A: LEARNER ASSESSMENT

1) Is there a standard process of assessing patient learning capabilities?	**Y/N**

2) Are patients' language/s recorded on file?	**YES**	**all languages**	**first language only**
	**NO**		

3) Is there a documented assessment of spoken English capability?	**YES**	**method:**
	**NO**	

4) Is there a documented assessment of literacy/numeracy level	**YES**	**method:**
	**NO**	

5) Are patient learning style preferences recorded (e.g. prefers action, face/face etc)	**YES**	**method:**
	**NO**	

Part B: PROGRAM STRUCTURE

6) Who has overall responsibility for patient education programs at this site?
7) How are the contributions/roles of different professionals co-ordinated?
8) What are the discrete identifiable education programs on site?

9) Do program/s have explicit written objectives?	**Y/N**
10) Do programs have quality review cycles?	**Y/N**

Part C: PROGRAM PROCESSES

C1: Personnel

11) What categories of patient educator does the site employ?

	**F/T**	**P/T**

12) Are there Aboriginal/TI employees working in the educational programs?	**Y/N**

C2: Documentation

13) Are records kept of patient education program/s?	**Y/N**
14) Is individual patient educational progress documented?	**Y/N**

C3: interpreter use

15) Are interpreters used here?	**YES**	**regularly**	**occasionally**	**hardly ever**
	**NO**			

16) Who is employed to interpret?				
**accredited professionals**	**family members**	**staff**	**other patients**	**all of these**

17) When non-accredited interpreters are employed, are they paid?	**Y/N**

18) How accessible/available are interpreters locally?

**easily accessed**	**available**	**difficult to access**	**unavailable**	**not sure**

19) Could you describe your most recent use of interpreter:

**Date:**	**Qualification:**	**Language:**

PART D: PROGRAM SETTING

20) Is there a space dedicated specifically to educational activities?
YES **Comment:**

NO **Comment:**

21) If yes, is there sufficient room for group work (e.g. family members)?
YES **Comment:**

NO **Comment:**

22) Is the educational setting differentiated or separated from the clinical surroundings?
YES **Comment:**

NO **Comment:**

23) If no specific educational space is available, where does education usually take place?

24) Are there readily accessible opportunities for self-guided education (e.g. internet, books, fact sheets etc
YES **Comment:**

NO **Comment:**
