# Supplementary material for: Study Protocol – Improving Access to Kidney Transplants (IMPAKT): A detailed account of a qualitative study investigating barriers to transplant for Australian Indigenous people with end-stage kidney disease
Source: BMC Health Serv Res. 2008 Feb 4;8:31. doi: 10.1186/1472-6963-8-31 (PMC2275237; doi:10.1186/1472-6963-8-31)
Supplement: Additional file 27 — PDF, Patient Educational Resources Checklist; A sheet used to record resources available for use in patient education including 'self serve' information. [file 1472-6963-8-31-S27.pdf]

EDUCATIONAL RESOURCES CHECKLIST IMPQ2

SITE ……………………………………………………….. DATE……………… Page No…….

TITLE	TYPE*	TOPIC*	DATE	LAN/GE	PRODUCED L/C	USE LEVEL 1-10	COMMENT

* See codes for **TYPE, TOPIC** over page
Produced either locally (L) or professional/commercial (C) (e.g. Baxter's material)
Use Level = on a scale of 1-10 where '1' means the resource is rarely used, and '10' is a resource that is used with almost every patient.

*CODES FOR RESOURCE TYPE

1	pamphlett/factsheet
2	flipchart
3	booklet
4	book
5	poster/chart
6	postcard/photo
7	video
8	CD/DVD
9	diagram/image
10	artwork
11	other
12	animated sequence

*CODES FOR TOPICS

G	Kidney disease: general introduction
AT	All Treatment types
HD	Haemodialysis
PD	Peritoneal Dialysis
Tx	Transplant
OD	Organ Donation
K	Kidneys and their function
KDI	Kidney disease specific issue (e.g. anaemia)
O	Other health including diabetes etc
