# Supplementary material for: Study Protocol – Improving Access to Kidney Transplants (IMPAKT): A detailed account of a qualitative study investigating barriers to transplant for Australian Indigenous people with end-stage kidney disease
Source: BMC Health Serv Res. 2008 Feb 4;8:31. doi: 10.1186/1472-6963-8-31 (PMC2275237; doi:10.1186/1472-6963-8-31)
Supplement: Additional file 28 — PDF, IMPAKT card sort transplant knowledge; Content of cards; these were transferred to coloured card, cut into strips and laminated. [file 1472-6963-8-31-S28.pdf]

IMPAKT: Cards for sorting

1. A person must have lots medical tests before they get a new kidney.

2. A person waiting for a new kidney must stay fit and healthy.

3. Sometimes a new kidney stops working.
   That person must go back to dialysis.

4. After a person gets a new kidney they must stay in hospital for a long time – up to 1 month.

5. The operation for a new kidney happens in a big city hospital in...
   (4 different cards included 4 city names)

6. Later on, after the new kidney settles down and everything is working well, a person with a new kidney might go home.

7. When a person has the operation to get a new kidney sometimes things go wrong.

8. My kidneys can get better.

9. A person with a new kidney can stop taking medicines.

10. When a person gets a new kidney there will be no more blood tests and no more kidney doctor visits.

11. Missing dialysis sessions helps a person to get a new kidney.

12. When a person gets called for a new kidney all their family can go with them.
